# Loss of TRPV2-mediated blood flow autoregulation recapitulates diabetic retinopathy in rats

**DOI:** 10.1172/jci.insight.155128

**Published:** 2022-09-22

**Authors:** Michael O’Hare, Gema Esquiva, Mary K. McGahon, Jose Manuel Romero Hombrebueno, Josy Augustine, Paul Canning, Kevin S. Edgar, Peter Barabas, Thomas Friedel, Patrizia Cincolà, Jennifer Henry, Katie Mayne, Hannah Ferrin, Alan W. Stitt, Timothy J. Lyons, Derek P. Brazil, David J. Grieve, J. Graham McGeown, Tim M. Curtis

**Affiliations:** 1 Wellcome-Wolfson Institute for Experimental Medicine and; 2 Centre for Biomedical Sciences Education, Queen’s University Belfast, Belfast, United Kingdom.

**Keywords:** Ophthalmology, Vascular Biology, Diabetes, Ion channels, Microcirculation

## Abstract

Loss of retinal blood flow autoregulation is an early feature of diabetes that precedes the development of clinically recognizable diabetic retinopathy (DR). Retinal blood flow autoregulation is mediated by the myogenic response of the retinal arterial vessels, a process that is initiated by the stretch‑dependent activation of TRPV2 channels on the retinal vascular smooth muscle cells (VSMCs). Here, we show that the impaired myogenic reaction of retinal arterioles from diabetic animals is associated with a complete loss of stretch‑dependent TRPV2 current activity on the retinal VSMCs. This effect could be attributed, in part, to TRPV2 channel downregulation, a phenomenon that was also evident in human retinal VSMCs from diabetic donors. We also demonstrate that TRPV2 heterozygous rats, a nondiabetic model of impaired myogenic reactivity and blood flow autoregulation in the retina, develop a range of microvascular, glial, and neuronal lesions resembling those observed in DR, including neovascular complexes. No overt kidney pathology was observed in these animals. Our data suggest that TRPV2 dysfunction underlies the loss of retinal blood flow autoregulation in diabetes and provide strong support for the hypothesis that autoregulatory deficits are involved in the pathogenesis of DR.

## Graphical abstract

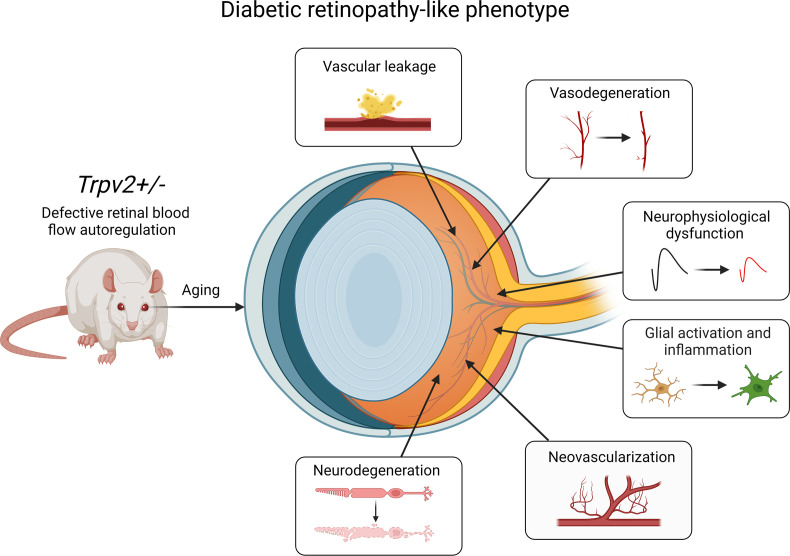

## Introduction

Diabetic retinopathy (DR) is a serious complication of diabetes and remains a major cause of irreversible vision loss and blindness worldwide (1, 2). Although good glycemic control, treatment for hypertension, and correction of dyslipidemia reduce the risk of DR, a substantial proportion of individuals still progress to the sight‑threatening complications of the disease (3). Available treatments for DR are limited to the advanced stages of the disease. Laser photocoagulation remains the mainstay treatment for proliferative DR (PDR) but is inherently destructive and creates the risk of permanent damage to peripheral vision (4). While intravitreal anti-VEGF treatments have become the standard of care in patients with diabetic macular edema (DME), not all individuals experience improvements in visual acuity, and others fail to respond or become refractory to treatment (5). With the global prevalence of diabetes increasing rapidly (6), there remains an unmet medical need for new treatment strategies, particularly those with efficacy in the early stages of the disease process. To enable this, it is crucial to gain a better understanding of the early pathophysiological events that contribute to the onset and progression of the disease.

It has been known for many years that changes in retinal blood flow precede the earliest clinical stages of DR (7). Loss of pressure autoregulation in the retina has been identified as a key mechanism that contributes to the disruption of retinal blood flow during early diabetes (8, 9). Retinal pressure autoregulation refers to the ability of the retina to keep total and regional blood flow constant, despite changes in arterial or intraocular pressure (IOP) (10). Impaired pressure autoregulation has been proposed to induce retinal injury in diabetes by raising retinal capillary pressures and causing shear-induced endothelial cell damage (11). Such changes would be expected to lead to several of the hallmarks of DR, including increased vascular permeability, capillary dropout, and ischemia-driven neovascularization (11). Recent work has shown that autoregulatory deficits predict the onset and progression of DR in subjects with type 1 diabetes, independently of their glycemic status (12). However, exactly why retinal pressure autoregulation breaks down in diabetes and whether impairment of this mechanism alone is sufficient to trigger DR-like pathology remains unclear.

Up to now, progress in elucidating the role of pressure autoregulation in the pathogenesis of DR has been hampered by a lack of knowledge on the basic molecular and cellular mechanisms that drive this process under normal physiological conditions. In common with other vascular beds, the myogenic response is considered the main mechanism underpinning pressure autoregulation in the retina (10). The myogenic response is defined as the intrinsic ability of small arterial vessels to constrict or dilate in response to increases or decreases in transmural pressure, respectively (13). Previous studies have shown that this primarily depends on the reaction of the vascular smooth muscle cells (VSMCs) in the vessel wall and is independent of any endothelial, neural, or humoral influences (14). We have made significant progress toward identifying the cellular signaling pathways that underpin the myogenic response in retinal arterioles (reviewed in ref. 15). Our data have shown that it is initiated by pressure‑induced stretching of the vessel wall, leading to the activation of mechanosensitive TRPV2 cation channels on the plasma membrane of the VSMCs (16). This causes depolarization of the cell membrane potential and an increase in Ca^2+^ influx through L- and T-type voltage–gated Ca^2+^ channels (VGCCs), ultimately leading to VSMC contraction and vasoconstriction (17, 18). Previous studies have observed defective myogenic constriction in diabetic individuals with no retinopathy (19), and it becomes more pronounced as the disease progresses (20). We have previously ruled out changes in VGCC activity and intracellular Ca^2+^ signaling mechanisms as being responsible for the diabetes-induced impairment of myogenic constriction in retinal arterioles (21, 22). These findings point to the defect most likely residing in the TRPV2 stretch‑sensing mechanism, and therefore, the ability of the retinal VSMCs to sense pressure changes across the vascular wall.

In the present study, we report that disruption of myogenic constriction in diabetic retinal arterioles is associated with the molecular and functional downregulation of TRPV2 channels and the loss of retinal VSMC stretch-activated cation currents. Moreover, we show that rats heterozygous for TRPV2, which are normoglycemic and normotensive, display defective myogenic and pressure autoregulatory responses in the retina and develop a range of microvascular lesions resembling those observed in DR. The retinas in these animals also exhibit inflammatory, gliotic, neurophysiological, and neurodegenerative changes similar to those observed in DR. Our findings suggest that loss of stretch-dependent TRPV2 channel activity underlies the impaired myogenic reactivity of retinal arterioles during diabetes. They also strongly support the hypothesis that impaired pressure autoregulation in the retina is causally linked to the onset and progression of DR.

## Results

### Defective myogenic regulation and TRPV2 channel function in diabetic retinal arterioles.

We began by assessing the myogenic response and TRPV2 channel expression and function in isolated retinal arterioles from nondiabetic and streptozotocin-induced (STZ-induced) diabetic rats of 3-month disease duration. The characteristics of the nondiabetic and diabetic rats used in this study are presented in Supplemental Figure 1 (supplemental material available online with this article; https://doi.org/10.1172/jci.insight.155128DS1). All the diabetic rats were hyperglycemic, as indicated by significantly elevated blood glucose and hemoglobin A1c (HbA1c) levels. As expected, body weight gain was lower in the diabetic rats than in the age-matched nondiabetic controls.

**Figure 1:**
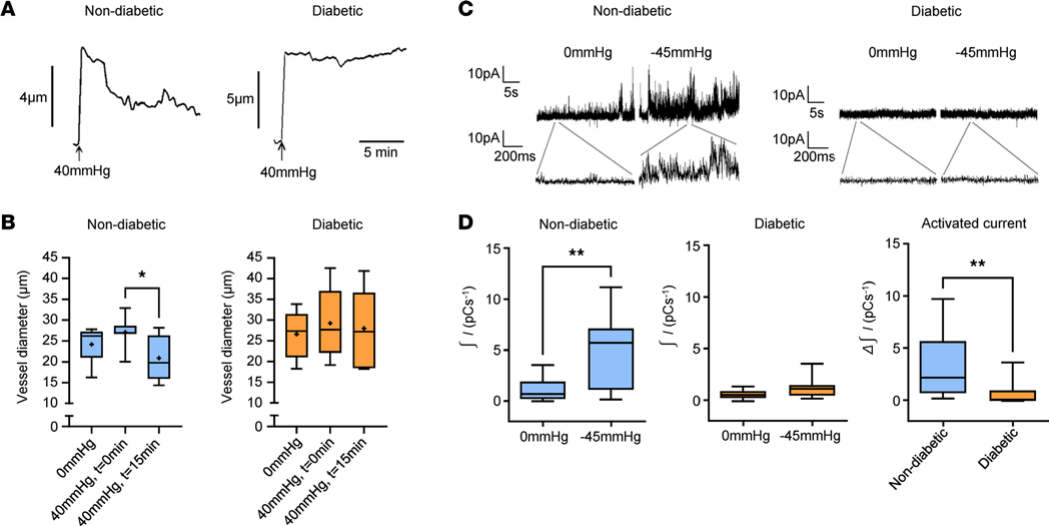
Defective myogenic signaling in retinal arterioles from diabetic rats of 3-month disease duration. (**A**) Representative pressure myograph recordings from retinal arterioles of nondiabetic and diabetic rats. (**B**) Box-and-whisker plots showing that the myogenic response is lost in retinal arterioles during experimental diabetes. *P < 0.05 based on repeated-measures ANOVA; *n* = 6–7 animals, *n* = 6–7 arterioles per group. (**C**) On-cell patch recordings of stretch-activated TRPV2 cation current activity in nondiabetic and diabetic retinal VSMCs. (**D**) Box-and-whisker plots showing that application of membrane stretch failed to activate TRPV2 cation currents in retinal arterioles from diabetic rats. **P < 0.01 based on Student’ *t* test; *n* = 6–7 animals, *n* = 13 VSMCs per group.

Pressure myography was used to assess the myogenic reactivity of isolated retinal arterioles from nondiabetic and diabetic rats. Pressurization of nondiabetic arterioles to 40 mmHg initially distended the vessel wall, but this was followed by an active constriction that reached steady-state levels after about 15 minutes (Figure 1, A and B). In contrast, following the pressure-induced dilation, no active constriction was observed in retinal arterioles from diabetic rats (Figure 1, A and B). Thus, like humans, the myogenic reactivity of retinal arterioles is compromised during early diabetes in experimental rats.

Stretch activation of TRPV2 channels plays an essential role in the myogenic constriction of retinal arterioles (16). We therefore studied the possibility that the loss of the myogenic response in diabetic retinal arterioles could be related to changes in stretch-dependent TRPV2 channel activity in these vessels. Stretch-induced TRPV2 currents were recorded in VSMCs from nondiabetic and diabetic retinal arterioles by applying negative pressure (–45 mmHg) to the patch pipette during on-cell patch clamp recordings. Previously, we have shown that the stretch-induced currents evoked using this protocol are eliminated in the presence of the TRPV2 channel antagonist, tranilast, or by preincubating the vessels with TRPV2 pore‑blocking antibodies (16). Following membrane stretch, TRPV2 current activity increased > 5-fold in retinal VSMCs from nondiabetic arterioles, while those from diabetic animals displayed no significant response (Figure 1, C and D). These data show that membrane stretch fails to activate TRPV2 current activity in retinal VSMCs from diabetic rats.

**Figure 2:**
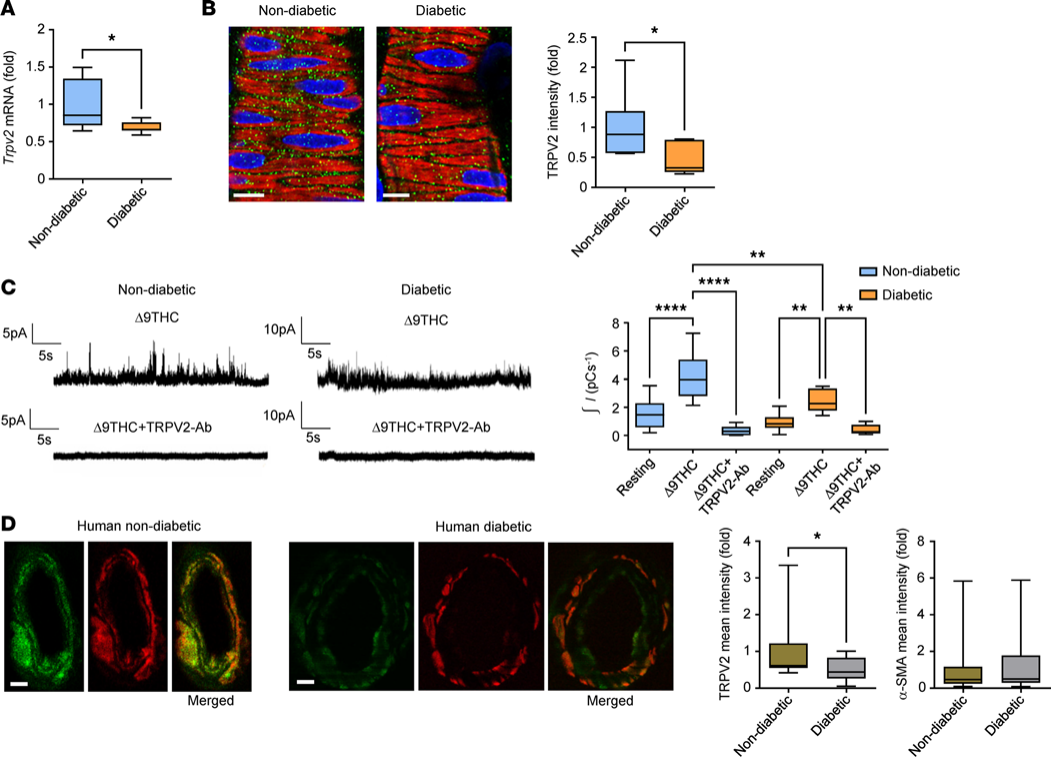
TRPV2 expression and function in diabetic retinal arterioles. (**A**) *Trpv2* mRNA expression in diabetic retinal arterioles expressed as a fold change from nondiabetic control values. *P < 0.05 based on Mann–Whitney *U* test; *n* = 9 animals per group. (**B**) Left, confocal images of nondiabetic and diabetic retinal arterioles embedded within retinal whole mount preparations and labeled for TRPV2 (green), α-SMA (red), and TO-PRO-3 nuclear stain (pseudocolored blue). Most of the TRPV2 staining localized to border regions between the adjacent retinal VSMCs, which we have previously shown correspond to the VSMC plasma membranes (16). Scale bars: 10 μm. Right, TRPV2 fluorescence intensity in diabetic retinal VSMCs expressed relative to nondiabetic controls. *P < 0.05 based on Mann–Whitney *U* test; *n* = 6 animals, *n* = 6 arterioles per group. (**C**) Left, on-cell patch-clamp recordings from nondiabetic and diabetic retinal arterioles with the TRPV2 agonist, Δ9-THC (10 μM; Tocris) included in the patch pipette. Traces in the lower panels are from arterioles that were preincubated for 1 hour with TRPV2 pore-blocking antibodies (1:100; Alomone, ACC-039). Right, box-and-whisker plots showing that Δ9-THC–induced currents were significantly smaller in diabetic compared with nondiabetic arterioles. **P < 0.01, ****P < 0.0001 based on 2-way ANOVA; *n* = 5–9 animals, *n* = 5–13 arterioles per group. (**D**) Left, confocal cross-sectional images of human nondiabetic and diabetic retinal arterioles labeled for TRPV2 (green) and α-SMA (red). Scale bars: 15 μm. Right, Summary data showing that TRPV2-associated immunofluorescence, but not α-SMA–associated immunofluorescence, was significantly lower in human diabetic retinal VSMCs. *P < 0.05 based on Mann–Whitney *U* test; *n* = 4 postmortem donors, *n* = 16 arterioles per group.

Quantitative PCR (qPCR) and IHC experiments were undertaken to examine whether changes in the gene and protein expression of TRPV2 channels might contribute to the loss of the myogenic response and stretch-dependent TRPV2 current in diabetic retinal arterioles. *Trpv2* mRNA transcript levels were reduced by 31% in isolated retinal arterioles from diabetic compared with nondiabetic rats (Figure 2A). TRPV2 immunoreactivity in retinal VSMCs from nondiabetic rats exhibited a punctate labeling pattern localized to plasmalemmal and cytosolic regions of the cells (Figure 2B). In retinal VSMCs from diabetic rats, TRPV2‑associated immunofluorescence was decreased by 55% (Figure 2B).

To confirm functional loss of TRPV2 channels on the plasma membrane of diabetic retinal VSMCs, on-cell patch clamp recordings were performed using pipette solutions containing the TRPV2 agonist, Δ9-tetrahydrocannabinol (Δ9-THC) (16, 23). In nondiabetic retinal VSMCs, Δ9-THC increased cation current activity in a manner similar to that observed with direct membrane stretch (Figure 2C). This drug also enhanced cation current activity in diabetic retinal VSMCs, but the magnitude of the response was markedly reduced (Figure 2C). In addition to being a potent TRPV2 agonist, Δ9‑THC has also been reported to activate TRPA1 and TRPV3 channels with moderate potency (24, 25). To exclude the possibility that our measurements were contaminated by these or other currents, experiments were repeated using arterioles preincubated with TRPV2-pore blocking antibodies (16, 26). Under these conditions, Δ9-THC failed to induce any significant current activation in retinal VSMCs from either group of animals (Figure 2C). Collectively, these findings show that diabetes downregulates the molecular and functional expression of TRPV2 channels in retinal VSMCs.

To investigate whether our findings in diabetic rats are relevant to human diabetes, TRPV2 protein expression was evaluated by semiquantitative immunofluorescent staining of postmortem retinal sections from nondiabetic and diabetic human subjects. TRPV2 was found to be highly expressed in the VSMC layer of nondiabetic human retinal arterioles, although the labeling was less punctate than in rats (Figure 2D). TRPV2-associated immunofluorescence was 50% lower in retinal VSMCs from diabetic donors (Figure 2D), consistent with our observations in diabetic rats. α-SMA immunoreactivity was similar in retinal VSMCs from both groups of subjects (Figure 2D).

### Retinal myogenic and pressure autoregulatory responses are lost in TRPV2 heterozygous rats.

It has yet to be established experimentally whether loss of the myogenic response, leading to failure of pressure autoregulation of blood flow in the retina, is capable of contributing directly to the development of the retinal lesions characteristic of DR. One way to address this issue would be through the development of a nondiabetic model of impaired retinal myogenic activity and pressure autoregulation. To this end, TRPV2 heterozygous rats (*Trpv2^+/–^*) were generated using CRISPR-Cas9 genome editing technology (Supplemental Figures 2 and 3). Homozygous TRPV2-KO rats were not viable and were reabsorbed in utero by E15.

**Figure 3:**
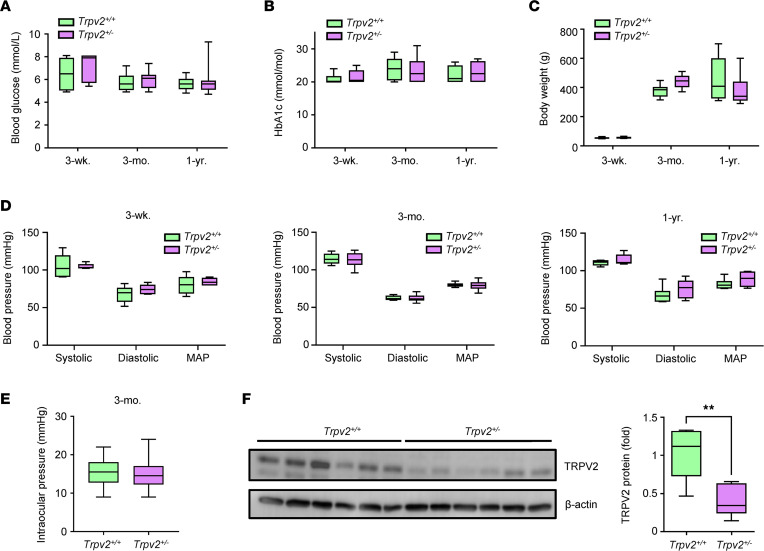
Characteristics of TRPV2 WT and heterozygous rats. (**A**–**C**) Box-and-whisker plots comparing blood glucose, HbA1c, and body weights of TRPV2 heterozygous rats with those of the TRPV2 WT controls at 3 weeks, 3 months, and 1 year of age. NS based on 2-way ANOVA; *n* = 6–17 animals per group for blood glucose, *n* = 6–10 animals per group for HbA1c, and *n* = 6–13 animals per group for weights. (**D**) Tail cuff plethysmography revealed no differences in systolic, diastolic, and mean arterial blood pressures (MAP) between TRPV2 WT and heterozygous rats at any of the time points examined. NS based on 2-way ANOVA; *n* = 6 animals per group. (**E**) Summary data showing that intraocular pressures were similar in TRPV2 WT and heterozygous rats at 3 months of age. NS based on Student’ *t* test; *n* = 7–8 animals per group. (**F**) Left, representative Western blot showing that TRPV2 protein levels were markedly lower in the retinas of TRPV2 heterozygous rats versus their WT counterparts. Each lane represents an individual animal. As expected, the TRPV2 band size was ~98 kDa. Right, quantification of Western blot data confirmed a reduction in TRPV2 protein levels in retinas from TRPV2 heterozygous rats. **P < 0.01 based on Mann–Whitney *U* test; *n* = 6 animals per group.

TRPV2 WT and heterozygous rats were normoglycemic up to 1 year of age, with blood glucose and HbA1c levels falling within the normal range (Figure 3, A and B). No statistical differences in body weights were observed at any time point between the 2 groups (Figure 3C). Both groups of animals exhibited normal blood and IOPs (Figure 3, D and E). Western blotting of retinal samples was performed to confirm reduced protein expression of TRPV2 in heterozygous rats. TRPV2 protein levels were 61% lower in the retinas of TRPV2 heterozygous versus WT rats (Figure 3F).

**Figure 4:**
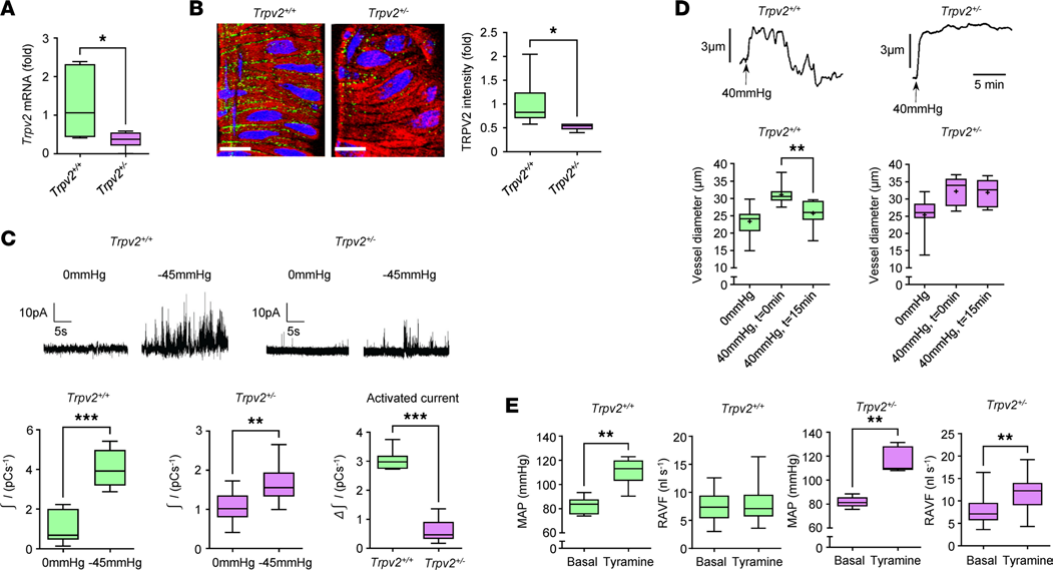
Impaired retinal myogenic signalling and blood flow autoregulation in TRPV2 heterozygous rats. (**A**) *Trpv2* mRNA expression in retinal arterioles from TRPV2 heterozygous rats expressed as a fold change from WT control values. *P < 0.05 based on Mann–Whitney *U* test; *n* = 6 animals per group. (**B**) Left, confocal images illustrating reduced TRPV2 protein expression (green) in retinal arterioles from TRPV2 heterozygous rats. Retinal VSMCs were colabeled for α-SMA (red) and nuclei stained using TO-PRO-3 (pseudocolored blue). Scale bars: 15 μm. Right, TRPV2 fluorescence intensity in retinal VSMCs from TRPV2 heterozygous animals expressed relative to TRPV2 WT controls. *P < 0.05 based on Mann–Whitney *U* test; *n* = 6 animals, *n* = 6 arterioles per group. (**C**) Top, on-cell patch recordings of stretch-activated TRPV2 cation current activity in retinal VSMCs from TRPV2 WT and TRPV2 heterozygous rats. Bottom, box-and-whisker plots showing that stretch-induced TRPV2 cation current activity was markedly reduced in retinal VSMCs from TRPV2 heterozygous rats. **P < 0.01, ***P < 0.001 based on Mann–Whitney *U* test; *n* = 6 animals, *n* = 7–10 VSMCs per group. (**D**) Top, pressure myograph traces from retinal arterioles of TRPV2 WT and TRPV2 heterozygous rats. Bottom, summary data showing that the myogenic reaction was completely absent in isolated retinal arterioles from TRPV2 heterozygous rats. **P < 0.01 based on repeated-measures ANOVA; *n* = 6–7 animals, *n* = 8-10 arterioles per group. (**E**) Pooled data comparing MAP and retinal arteriolar volumetric flow (RAVF) in TRPV2 WT and TRPV2 heterozygous rats under basal conditions and following i.v. injection of tyramine. **P < 0.01 based on Mann–Whitney *U* test; *n* = 6 animals per group for MAP; *n* = 6 animals, *n* = 19–23 arterioles per group for RAVF. Basal RAVF was not significantly different between the 2 groups of animals (*P* > 0.05; Mann–Whitney *U* test).

Next, experiments were undertaken to establish whether retinal myogenic signaling is defective in TRPV2 heterozygous rats. We firstly compared the gene, protein, and stretch-dependent functional expression of TRPV2 channels in retinal arterioles between the 2 groups of animals. *Trpv2* gene expression was decreased by 72% in retinal arterioles from the heterozygous rats when compared with the WT animals (Figure 4A). In heterozygous rats, TRPV2 protein expression in the retinal VSMCs was about half that of the WT animals, as assessed by confocal immunofluorescence microscopy (Figure 4B). TRPV2 cation current activity was also substantially reduced in the retinal VSMCs of heterozygous rats, although some current activation was still observed upon membrane stretch (Figure 4C). Subsequent pressure myography experiments showed that retinal arterioles of WT rats constricted normally in response to an increase in intraluminal pressure (Figure 4D). In contrast, the myogenic reaction was completely absent in retinal arterioles of heterozygous rats (Figure 4D). Transcript levels for other ion channels that are known to regulate the retinal myogenic response (*Cacna1c*, *Cacna1g*, *Ryr2*, *Itpr1*, *Itpr2*, *Kcna5*, and *Kcnma1*; ref. 15) were similar in arterioles isolated from the 2 groups of animals (Supplemental Figure 4). Hence, the complete loss of myogenic function in the heterozygous vessels could not be attributed to alterations in the expression of these genes.

To confirm that the loss of myogenic function in retinal arterioles from TRPV2 heterozygous rats results in pressure autoregulatory deficits similar to those previously reported in the diabetic retina (8, 27), in vivo blood flow studies were performed. Retinal arteriolar volumetric flow (RAVF) was measured in TRPV2 WT and heterozygous rats under basal conditions and following a tyramine-induced increase in blood pressure. Tyramine was selected for these studies because it has no effect on IOP and does not directly stimulate the retinal vessels, as they lack sympathetic innervation (9, 28). RAVF and in vivo arteriolar diameters were similar for both groups of animals under basal conditions (Figure 4E and Supplemental Figure 5). I.v. administration of tyramine (1 mg/kg) elevated mean arterial pressure (MAP) to a similar degree in both TRPV2 WT and heterozygous animals (Figure 4E). RAVF remained unchanged in WT rats following the tyramine-induced elevation of MAP but increased by 45% in the heterozygous rats (Figure 4E), indicating a loss of autoregulatory control.

Overall, these data demonstrate that TRPV2 heterozygous rats offer a useful nondiabetic model to study the consequences of impaired myogenic activity and pressure autoregulation in the retina.

### Nondiabetic TRPV2 heterozygous rats develop DR-like vascular defects and retinal neovascularization.

DR is characterized by progressive vascular changes in the retina, including increased vascular leakage, the formation of acellular capillaries, and retinal neovascularization (1, 4, 11). To determine whether such changes might be connected to a loss of retinal pressure autoregulation, we compared retinal vascular permeability and morphology in TRPV2 WT and heterozygous rats up to 1 year of age.

**Figure 5:**
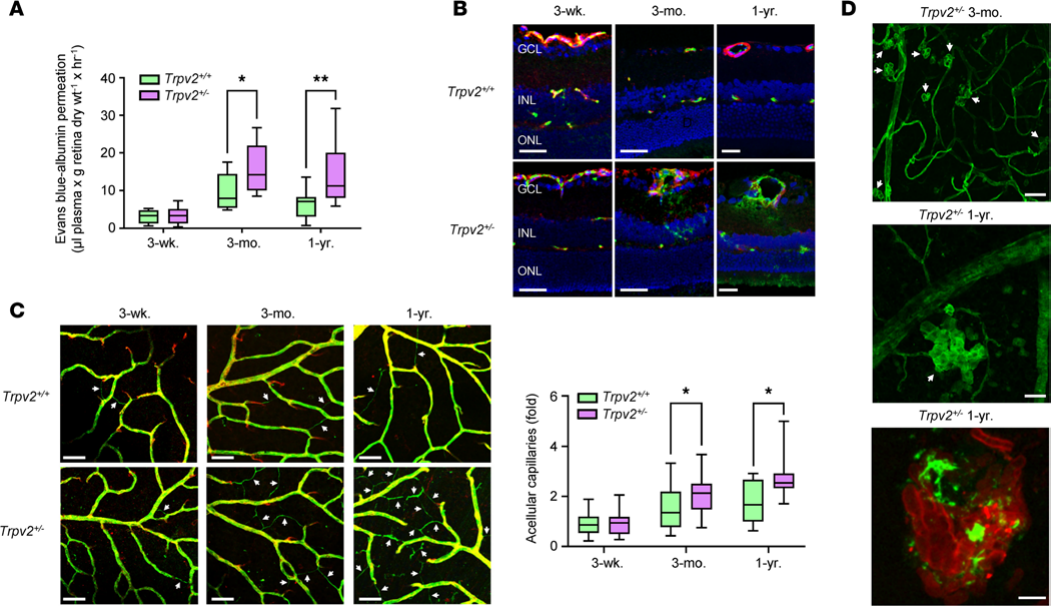
Retinal vascular defects in TRPV2 heterozygous rats. (**A**) Retinal Evans blue leakage in TRPV2 WT and heterozygous rats at 3 weeks, 3 months, and 1 year of age. *P < 0.05, **P < 0.01 based on 2-way ANOVA; *n* = 8–10 animals per group. (**B**) Representative confocal images showing retinal sections from TRPV2 WT and heterozygous rats for each of the 3 age groups labeled for albumin (green), isolectin-B4 (red), and DAPI nuclear stain (blue). Marked albumin leakage was apparent in retinal sections from TRPV2 heterozygous rats at 3 months and 1 year of age. Scale bars: 50 μm (3 weeks and 3 months) and 25 μm (1 year); GCL, ganglion cell layer; INL, inner nuclear layer; ONL, outer nuclear layer. (**C**) Left, confocal images of retinal whole mount preparations from TRPV2 WT and heterozygous rats at 3 weeks, 3 months, and 1 year of age labeled for collagen IV (green) and isolectin-B4 (red) focused at the plane of the superficial vascular plexus. Acellular capillaries, evident as degenerate vascular segments positive for collagen IV and negative for isolectin-B4, are denoted by white arrows. Scale bars: 50 μm. Right, box-and-whisker plots showing that acellular capillary formation was significantly increased in retinas from TRPV2 heterozygous animals at 3 months and 1 year of age. *P < 0.05 based on 2-way ANOVA; *n* = 6 animals, *n* = 6 retinas per group. (**D**) Top and middle, isolectin-B4–labeled retinal whole mount preparations illustrating angiogenic tuft formation (white arrows) in TRPV2 heterozygous rats at 3 months and 1 year of age. Scale bars: 50 μm. Bottom, high-magnification image of a retinal angiogenic tuft in a TRPV2 heterozygous rat at 1 year colabeled for RUNX1 (pseudocolored green) and isolectin-B4 (red). Scale bar: 10 μm.

The Evans blue technique was used to measure vascular protein leakage into the retina (29). No difference in retinal vascular permeability was observed in TRPV2 WT and heterozygous rats 3 weeks after birth (Figure 5A). By 3 months, however, Evans blue extravasation was significantly increased in TRPV2 heterozygous retinas — an effect that was sustained after 1 year (Figure 5A). As an additional approach to assessing inner blood retinal barrier breakdown, retinal sections were colabeled with an anti-albumin antibody and isolectin-B4 to stain the retinal vasculature. In TRPV2 WT retinas, albumin was only detected within the retinal blood vessels, irrespective of the animals age (Figure 5B). Consistent with our Evans blue studies, no albumin extravasation was seen in retinal sections from 3-week-old TRPV2 heterozygous rats, whereas significant leakage was evident in those from the 3-month-old and 1‑year-old animals (Figure 5B). This albumin leakage was focal in nature and mainly localized to perivascular regions of the retinal neuropile (Figure 5B).

The formation of acellular capillaries in the retina results from the death of the vascular component cells, leaving behind naked basement membrane tubes (30). In retinal flatmount preparations, acellular capillaries are easily identifiable as thin strands that are positive for the vascular basement membrane marker, collagen IV, and negative for the endothelial cell marker, isolectin-B4 (31). Using this technique, we detected no difference in the numbers of acellular capillaries in 3-week-old TRPV2 WT and heterozygous retinas (Figure 5C). However, at 3 months and 1 year of age, acellular capillary formation was significantly greater in TRPV2 heterozygous retinas when compared with those of their WT counterparts (Figure 5C).

Retinal neovascularization is the major pathological feature of PDR (2). It is caused by the hypoxia-induced upregulation of angiogenic growth factors and inflammatory cytokines that results from the formation of acellular capillaries and capillary nonperfusion (32, 33). In isolectin-B4–stained retinal flatmounts, we found that TRPV2 heterozygous rats develop spontaneous neovascular tufts in the superficial plexus (Figure 5D). These were observed in all retinas from the 1-year-old animals and sporadically in those at the 3-month time point (2 out of 6 animals). In both groups of animals, the areas of neovascularization were relatively sparse and patchy in their topographic distribution. In the 1-year-old animals, for example, retinal coverage by neovascular tufts averaged 2% and varied from 0.3% to 5.7% among animals. Angiogenic tufts stained positive for runt-related transcription factor 1 (RUNX1), a selective marker for proliferating endothelial cells and aberrant angiogenesis in the retina (34). Neovascular tufts were not detected in retinas from any of the WT groups or those of the 3-week-old heterozygous rats (Figure 5D).

### TRPV2 heterozygous retinas display glial cell and inflammatory changes.

Glial cell dysfunction and low-grade inflammatory signaling in the retina have been linked to the pathogenesis of DR (33, 35, 36). We were, therefore, interested in exploring whether similar changes are recapitulated in the retinas of TRPV2 heterozygous rats.

**Figure 6:**
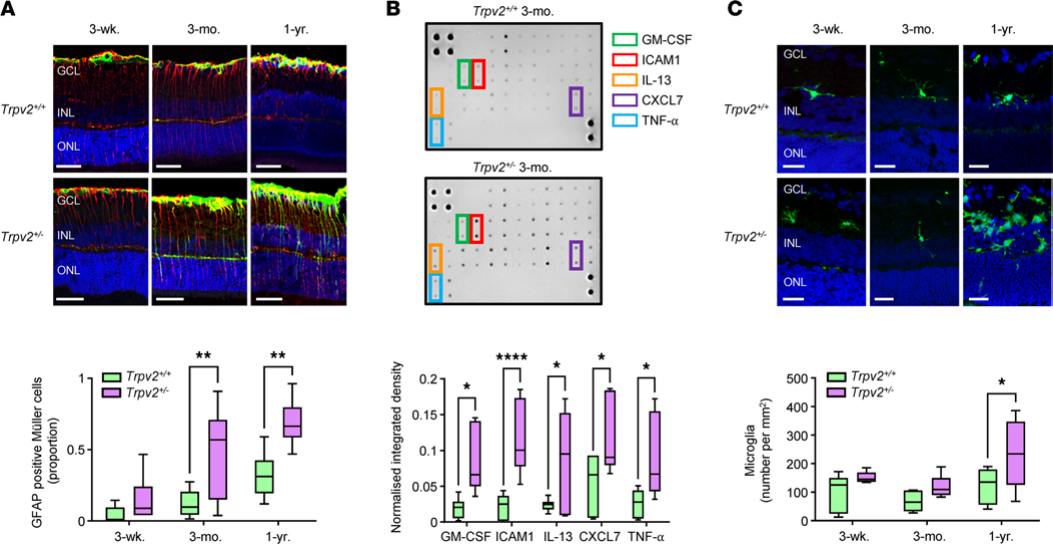
Glial and inflammatory changes in TRPV2 heterozygous retinas. (**A**) Top, representative confocal images of retinal sections from TRPV2 WT and heterozygous rats at 3 weeks, 3 months, and 1 year of age labeled for GFAP (green), vimentin (red; Müller cell marker), and TO-PRO-3 nuclear stain (pseudocolored blue). Scale bars: 50 μm. GCL, ganglion cell layer; INL, inner nuclear layer; ONL, outer nuclear layer. Bottom, box-and-whisker plots showing that the proportion of GFAP^+^ Müller cell fibers was significantly increased in TRPV2 heterozygous retinas at 3 months and 1 year of age. **P < 0.01 based on 2-way ANOVA; *n* = 6 animals, *n* = 6 retinas per group. (**B**) Top, representative cytokine array images from TRPV2 WT and heterozygous retinas at 3 months. Each cytokine is spotted in duplicate, and the location of GM-CSF, ICAM-1, IL-13, CXCL7, and TNF-α is denoted using colored boxes. Bottom, quantitative analysis of the arrays was performed by densitometry, and values were normalized to positive control spots on each membrane. ****P < 0.0001, *P < 0.05 based on 2-way ANOVA; blots were performed in triplicate with 3 different biological pools of retinas for each genotype; *n* = 18 animals per group in total, *n* = 6 animals per replicate. (**C**) Top, Iba1-immunolabeled microglia (green) in retinal sections from TRPV2 WT and heterozygous rats at 3 weeks, 3 months, and 1 year of age. Nuclei were labeled with TO-PRO-3 (pseudocolored blue). Scale bars: 25 μm. Bottom, summary data showing that microglia numbers were markedly increased in retinas from TRPV2 heterozygous rats at 1 year of age. *P < 0.05 based on 2-way ANOVA; *n* = 5 animals, *n* = 5 retinas per group.

During DR, Müller glia undergo a reactive gliosis, which is characterized by the upregulation of glial fibrillary acidic protein (GFAP) and the production of inflammatory mediators that contribute to the induction of retinal inflammation (36, 37). To visualize Müller cell gliosis, transverse retinal cryosections from TRPV2 WT and heterozygous rats were colabeled for GFAP and the Müller cell marker, vimentin. No significant difference in GFAP expression was observed in the 3-week-old animals (Figure 6A). Indeed, in both the WT and heterozygous retinas, GFAP immunoreactivity was mainly localized to the retinal astrocyte layer and largely absent in vimentin^+^ Müller cell processes (Figure 6A). By 3 months, however, Müller cell gliosis was evident in the TRPV2 heterozygous but not the WT retinas, as indicated by a marked increase in the proportion of GFAP^+^ Müller cell fibers (Figure 6A). After 1 year, this effect became more pronounced (Figure 6A).

Since Müller cell gliosis develops in TRPV2 heterozygous retinas, we proceeded to investigate whether this is associated with a concomitant upregulation of retinal inflammatory cytokine levels. These experiments were limited to the analysis of 3-month-old TRPV2 WT and heterozygous retinas using a rat cytokine antibody array panel. Of the inflammatory factors represented on the array, GM-CSF, ICAM-1, IL-13, CXCL7, and TNF-α were found to be significantly upregulated in the retinas of the TRPV2 heterozygous rats (Figure 6B), with all other factors remaining unchanged. The full results of the cytokine arrays are presented in Supplemental Table 1.

To further analyze inflammatory changes, retinal cryosections were immunolabeled with the microglial marker, ionized calcium binding adaptor molecule 1 (Iba1). In 3-week-old and 3-month-old animals, no significant differences in microglial numbers between WT and heterozygous retinas could be detected (Figure 6C). Furthermore, in these sections, the microglia displayed a dendritic morphology, suggesting that they were not reactive (Figure 6C). However, by 1 year of age, the TRPV2 heterozygous retinas had many more microglia than those of the WT animals, and most had transformed to a more amoeboid, reactive shape (Figure 6C). Infiltration of microglia into the outer retinas of the 1-year-old heterozygous rats was also seen, and this was not evident in the retinal sections from WT animals (Figure 6C).

### Neurophysiological and neurodegenerative changes in TRPV2 heterozygous retinas.

Diabetes not only causes retinal microvascular disease and glial cell dysfunction, but it also triggers early electrophysiological defects and longer-term neurodegenerative changes in the retina (38). Thus, we sought to determine whether retinal neuronal dysfunction and cell loss occurs in TRPV2 heterozygous animals.

**Figure 7:**
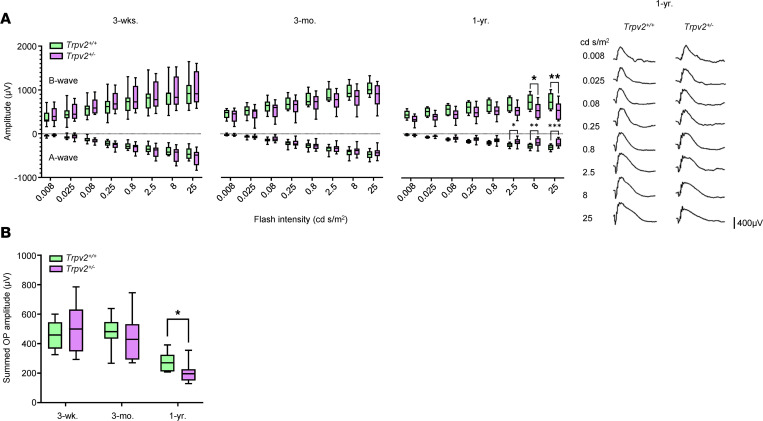
Retinal neurophysiological changes in TRPV2 heterozygous rats. (**A**) Left, box-and-whisker plots comparing ERG a- and b-wave amplitudes for TRPV2 WT and heterozygous rats at 3 weeks, 3 months, and 1 year of age. *P < 0.05, **P < 0.01, ***P < 0.001 based on 2-way ANOVA. *n* = 6–7 animals per group. Right, representative scotopic ERG waveforms at a range of stimulus intensities for a TRPV2 WT and heterozygous rat at 1 year. (**B**) Summary data showing that summed oscillatory potential amplitudes for the TRPV2 heterozygous rats were reduced in amplitude at 1 year of age but not at earlier time points of 3 weeks and 3 months. *P < 0.05 based on 2-way ANOVA. *n* = 6–7 animals per group.

Reductions in electroretinogram (ERG) a-wave and b-wave, and summed oscillatory potential (OP) amplitudes, have been reported in diabetic patients and experimental animal models of diabetes (39–44). Our data show no differences in ERG a- or b-wave amplitudes between TRPV2 WT and heterozygous animals at 3 weeks or 3 months of age (Figure 7A). At 1 year, however, both components of the ERG waveform were significantly diminished in TRPV2 heterozygous rats at high light intensities (≥2.5 cd·s/m^2^; Figure 7A). The amplitudes of the summed OPs were also decreased in the TRPV2 heterozygous animals at 1 year of age but not at the earlier time points of 3 weeks and 3 months (Figure 7B). No change was seen in a-wave or b-wave, or in summed OP implicit times, in TRPV2 heterozygous rats at any time point (Supplemental Figure 6). Pupil diameters were not different between WT and heterozygous rats under the conditions used for ERG recording, suggesting that retinal illuminance did not differ significantly between the 2 groups of animals (Supplemental Figure 6).

**Figure 8:**
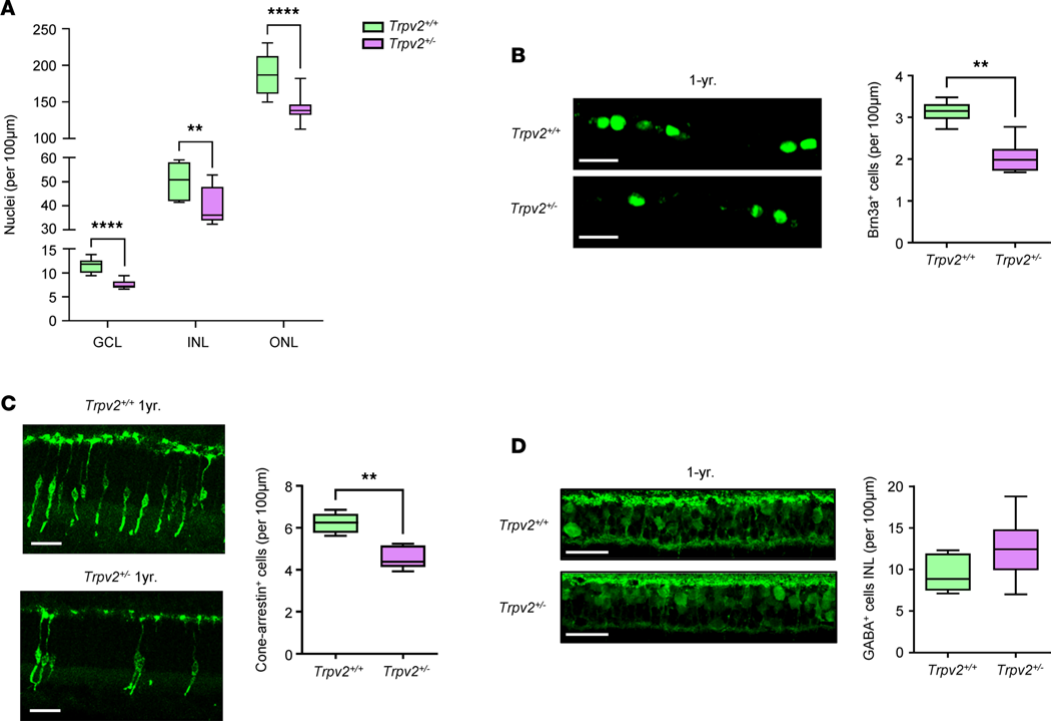
Retinal neurodegenerative changes in TRPV2 heterozygous rats. (**A**) Box-and-whisker plots showing the numbers of cell nuclei in the ganglion cell layer (GCL), inner nuclear layer (INL), and outer nuclear layer (ONL) of 1-year-old TRPV2 WT and heterozygous rats. **P < 0.01, ****P < 0.0001 based on Mann–Whitney *U* tests; *n* = 8–12 animals, *n* = 8–12 retinas per group (**B**) Left, retinal sections from a TRPV2 WT and heterozygous rat at 1 year of age focused on the GCL and processed for Brn3a immunoreactivity. Scale bars: 25 μm. Right, summary data comparing numbers of Brn3a^+^ retinal ganglion cells between the 2 genotypes. **P < 0.01 based on Mann–Whitney *U* test; *n* = 6 animals, *n* = 6 retinas per group. (**C**) Left, confocal images of cone arrestin immunoreactivity at the ONL for a TRPV2 WT and heterozygous rat at 1 year of age. Scale bars: 25 μm. Right, box-and-whisker plots showing that cone cell numbers were significantly reduced in TRPV2 heterozygous rat retinas. **P < 0.01 based on Mann–Whitney *U* test; *n* = 5 animals, *n* = 5 retinas per group. (**D**) Left, retinal sections from a TRPV2 WT and heterozygous rat at 1-year of age focused on the inner nuclear layer and processed for GABA immunoreactivity. Scale bars: 25 μm. Right, summary data showing that numbers of GABAergic amacrine cells remained unchanged in retinas from TRPV2 heterozygous rats. NS based on Mann–Whitney *U* test; *n* = 6 animals, *n* = 6 retinas per group.

To investigate neurodegenerative changes, the numbers of nuclei in the ganglion, inner nuclear layer, and outer nuclear layer were compared in TO-PRO-3–stained retinal sections from TRPV2 WT and heterozygous animals at 1 year of age. The number of cell nuclei was decreased in all nuclear layers of the TRPV2 heterozygous rats (Figure 8A). Neurodegeneration in the diabetic retina is characterized by the loss of specific neuronal cell populations, including retinal ganglion cells (RGCs), GABAergic amacrine cells, and cone photoreceptors (38, 45). Numbers of RGCs and cone photoreceptors were significantly reduced in retinal sections from the TRPV2 heterozygous versus WT rats (Figure 8, B and C). No differences were observed, however, in the numbers of GABAergic amacrine cells (Figure 8D). Retinal cell nuclei, RGC, and cone photoreceptor numbers were similar in 3-week-old TRPV2 WT and heterozygous retinas (Supplemental Figure 7), suggesting that the changes seen at 1 year were consequences of neurodegeneration rather than abnormal retinal development.

### No evidence of kidney pathology in TRPV2 heterozygous rats.

In addition to DR, impaired pressure autoregulation has been proposed to play an important role in the development of diabetic nephropathy (DN) (46). Unlike the retina, however, TRPV4 rather than TRPV2 channels have been proposed as the main mechanosensors that underlie myogenic autoregulation in the kidney (47). We, therefore, hypothesized that pathological lesions characteristic of DN should not develop in the kidneys of TRPV2 heterozygous rats. IHC staining of kidney sections confirmed previous reports that TRPV2 localizes most strongly to the renal tubules (Supplemental Figure 8) (48). TRPV2-associated immunofluorescence was 33% lower in the kidney sections from the TRPV2 heterozygous rats when compared with those of the WT group (Supplemental Figure 8). Kidney weights (adjusted to body weight) and renal morphology (as assessed by H&E staining) appeared normal in both TRPV2 heterozygous and WT rats up to 1 year of age (Supplemental Figure 8). These findings confirm that the kidneys of TRPV2 heterozygous rats have no obvious structural abnormalities.

## Discussion

It has been known since the 1980s that pressure autoregulation of retinal blood flow is disrupted in diabetic patients prior to the onset of clinical retinopathy (49). However, up to now, the mechanisms responsible have not been identified, and the relevance of this observation to the underpinning pathogenesis and progressive nature of DR has remained poorly understood. Data from the current study suggest that pressure autoregulatory deficits in the diabetic retina are caused by failure of the myogenic response resulting from the loss of stretch‑dependent TRPV2 current activity on the retinal VSMCs. Moreover, we have demonstrated that TRPV2 heterozygous rats, which exhibit impaired myogenic responses and autoregulatory function in the retina, develop a range of progressive microvascular, glial, and neuronal abnormalities that strongly resemble those observed in DR. These findings support the view that loss of pressure autoregulation alone may be sufficient to explain the initiation and progression of many of the retinal lesions observed in DR.

Our previous evidence that TRPV2 is the principal channel underlying stretch-dependent cation currents and myogenic constriction in retinal arterioles was based on studies using pharmacological and blocking-antibody approaches (16). We have demonstrated that inhibition of TRPV2 using tranilast or specific pore-blocking antibodies prevented the induction of stretch‑induced cation currents and myogenic tone development in these vessels (16). This study further substantiates these findings by showing that retinal arterioles from TRPV2 heterozygous rats display reduced stretch-activated cation current activity and a loss of myogenic reactivity. We were surprised to find that the myogenic response was completely absent in retinal arterioles from heterozygous rats, considering that these vessels still retained some stretch‑dependent TRPV2 current activity. This apparent discrepancy, however, could be explained if the pressure-induced activation of TRPV2 in heterozygous vessels was insufficient to produce enough depolarization, and hence voltage‑gated Ca^2+^ influx, to trigger retinal VSMC contraction. In addition to extending our previous work linking TRPV2 with retinal arteriolar myogenic signaling, this study is the first to our knowledge to demonstrate experimentally the involvement of these channels in mediating retinal pressure autoregulation in vivo. Specifically, we found that TRPV2 heterozygous rats failed to autoregulate their retinal blood flow in response to a tyramine-induced elevation of blood pressure. These data provide an important step forward in our understanding of the physiological mechanisms responsible for controlling blood flow in the retina. They also add to the growing literature suggesting that TRP channels play a key role in mediating pressure autoregulatory responses in vivo, with TRPM4 and TRPV4 previously being implicated in cerebral and renal blood flow autoregulation, respectively (47, 50). All our work thus far has focused on the involvement of TRPV2 channels in the myogenic reactivity of the larger arterioles in the retina. It is worth noting, however, that these channels are also expressed on the retinal VSMCs of the transitional arterioles, as well as the pericytes of the retinal capillaries (Supplemental Figure 9). Future work should be directed toward determining the role of these channels in the detection and transduction of mechanical stimuli in these vessels.

Although several studies have provided evidence that the myogenic reactivity of retinal blood vessels is compromised during diabetes (12, 19, 20), this study is the first to our knowledge to elucidate the underlying pathophysiological mechanisms. Our data indicate that the diabetes-induced impairment of myogenic tone is related to a complete loss of stretch-dependent TRPV2 current activity on the retinal VSMCs. Mechanistically, this effect of diabetes appears to be mediated, at least in part, through TRPV2 downregulation at the mRNA, protein, and functional levels. Nonetheless, since diabetic retinal VSMCs still possess some functional TRPV2 channels on their plasma membrane (as indicated by current responses to Δ9-THC), this cannot fully account for the complete loss of stretch‑dependent TRPV2 current activity. Our data, therefore, imply that diabetes may also act to uncouple signaling mechanisms linking membrane stretch to TRPV2 channel activation. It has yet to be determined how TRPV2 is activated by membrane stretch, with activation via direct mechanical (51) and indirect cell signaling (52) being suggested. A clearer knowledge of the mechanisms linking the activation of this channel to membrane stretch will be required to fully understand how diabetes disrupts stretch-dependent TRPV2 current in retinal VSMCs. The experiments involving TRPV2 heterozygotes indicate, however, that downregulation of TRPV2 expression to levels similar to those seen in diabetes is adequate to explain the loss of the myogenic response and pressure autoregulation in the retina. The demonstration that TRPV2 is both strongly expressed in the VSMCs of human retinal arterioles and is downregulated in human diabetes suggests that similar pathophysiological processes may underpin the loss of the retinal myogenic response and pressure autoregulation in both experimental and human diabetes.

TRPV2 heterozygous rats developed retinal vascular lesions reminiscent of those seen during DR. This included increased vascular leakage, acellular capillary formation, and the growth of neovascular complexes in the superficial layer of the retina. All these changes could be explained by the inability of these animals to autoregulate their retinal blood flow in response to fluctuations in arterial or IOP. Loss of retinal pressure autoregulation would be expected to cause overperfusion of the retinal capillaries, leading to disruption of the inner blood retinal barrier and capillary damage (11, 53). The progressive loss of retinal capillaries that then ensues may subsequently drive neovascularization through the induction of retinal ischemia and the upregulation of proinflammatory and angiogenic cytokines. Our findings support this sequence of events, given that neovascularization was only seen consistently in animals at 1 year of age when marked drop out of retinal capillary beds had occurred. While further studies are needed to identify the exact molecules responsible for triggering retinal neovascularization in TRPV2 heterozygous rats, our cytokine array data reveal increased expression of several factors that could play a role. This included GM-CSF, ICAM-1, IL-13, CXCL7, and TNF-α, all of which have been implicated in promoting angiogenesis in various tissues and organs under physiological and pathophysiological conditions (54–58). Notably, diabetic rodent models display similar levels of capillary degeneration to those observed in TRPV2 heterozygous rats (see, for example, ref. 59), but do not exhibit retinal neovascularization (11). It has previously been argued that the lack of retinal neovascularization in diabetic rodents results from both the short duration of diabetes and short lifespan of the animals (4, 60). However, our findings in TRPV2 heterozygous rats suggest that other factors may also be involved. Previous work from our own laboratories has shown that the diabetic milieu itself can suppress angiogenic signaling in the retina (61), which could explain the absence of neovascularization in diabetic models when compared with nondiabetic TRPV2 heterozygous rats.

Over recent years, glial cell activation, ERG dysfunction, and neuronal cell apoptosis have been established as prominent features of DR in both patients and experimental animal models of diabetes (38). While studies are ongoing, evidence to date has suggested that these changes arise due to the induction of oxidative stress, the accumulation of extracellular glutamate, and a decrease in the expression of neuroprotective factors within the diabetic retina (62). What has remained less clear, however, is whether the glial and neuronal abnormalities observed during DR result from the direct effects of diabetes on the retinal neuropile or develop as a secondary consequence of the early microvascular and hemodynamic changes associated with this disease (38, 62). Although our study does not directly address this question, our findings in TRPV2 heterozygous rats suggest that impairment of retinal blood flow autoregulation alone is sufficient to cause the development of glial and neuronal cell changes characteristic of DR. A recognized limitation of our study was the use of a global TRPV2 heterozygous rat model. In addition to being strongly localized to retinal VSMCs, TRPV2 expression has also been reported in RGCs, photoreceptor axons, and retinal pigment epithelial (RPE) cells (63–66). Thus, we cannot fully exclude the possibility that reduced TRPV2 expression in these cell types contributed to the ERG deficits and neurodegenerative changes observed in TRPV2 heterozygous rats at 1 year of age. Although several studies have reported that TRPV2 depletion has no direct impact on neuronal cell survival (67, 68), it is conceivable that the outer retinal deficits that occur in TRPV2 heterozygous rats may develop, at least in part, due to the loss of these channels in the RPE. In this regard, Ca^2+^ signaling via TRPV2 has been linked with VEGF secretion by the RPE (65) and RPE-derived VEGF is known to play an important role in maintaining photoreceptor cell survival in the retina (69, 70).

Impaired renal autoregulation has been implicated in the development of diabetic kidney injury (46). In our study, we observed no obvious renal phenotype in TRPV2 heterozygous rats up to 1 year of age. These results were perhaps not surprising, given that TRPV4 rather than TRPV2 has been identified as the main mechanosensor underlying myogenic autoregulation in the kidney (47). Our IHC data confirm previous reports that TRPV2 is highly expressed in epithelial cells of the renal tubules (48). To the best of our knowledge, there has been no research to date that has specifically investigated the physiological role of TRPV2 in the kidney, but our findings provide strong impetus for such work in the future.

In summary, we have presented data that strongly support the view that myogenic and pressure autoregulatory deficits in the diabetic retina result from the loss of stretch-dependent TRPV2 current activity in retinal VSMCs. We have also demonstrated that TRPV2 heterozygous rats lose their ability to autoregulate their retinal blood flow in response to blood pressure changes and develop retinal lesions similar to those observed during DR. It will be interesting in the future to examine the effects of superimposing diabetes on these animals, enabling an assessment of the extent to which other changes associated with the diabetic milieu exert additive or compensatory effects on retinal disease development. Overall, our findings provide the first direct preclinical evidence to our knowledge to support the hypothesis that autoregulatory deficits contribute to the pathogenesis of DR. They also suggest that targeting of TRPV2 dysfunction should be further explored for its potential to prevent the onset and progression of DR.

## Methods

### Diabetic rat model.

Male Sprague Dawley rats (200–250 g; Envigo) were i.p. injected with 65 mg/kg of STZ (Sigma-Aldrich, S0130) prepared in 0.1 mol/L sodium citrate buffer (pH 4.5). Control animals were sham injected with citrate buffer only. One week after STZ injection, blood glucose was determined using the tail prick method and a Breeze 2 glucometer (Bayer Scientific). Animals with blood glucose levels above 15 mmol/L were considered diabetic and selected for further study. All animals were weighed on a weekly basis and maintained and fed in the Biological Services Unit (BSU) at Queen’s University Belfast under a 12-hour light/dark (L/D) cycle (40). At 3 months of diabetes duration, blood glucose, and HbA1c levels (PTS Diagnostics A1CNow+ kit) were measured and the animals sacrificed by CO_2_ asphyxiation.

### TRPV2 heterozygous rat model.

Heterozygous *Trpv2^+/–^* rats were generated by SAGE labs. The rats were produced on a Sprague Dawley background using CRISPR-Cas9 genome editing with a guide RNA targeting exon 5 of the *Trpv2* gene. A 115 bp deletion was introduced that produced a premature stop codon in exon 5 (Supplemental Figure 2). The breeding of heterozygous TRPV2 rats yielded no viable homozygous TRPV2-KO animals. Heterozygous breeder rats were paired to generate the TRPV2 heterozygous (+/–) and WT (+/+) rats used in this study. Animals were maintained in SPF conditions (12-hour L/D cycle), and both male and female rats were used for experimentation. During the light phase, cage lighting levels were 262 ± 64 lux (mean ± SD; LX1330B Digital Light Meter; Dr.meter). Rats were studied at 3 weeks, 3 months, and 1 year of age to investigate age-dependent physiological and structural changes in the retina. Animal weights, blood glucose, and HbA1c levels were evaluated prior to sacrifice by CO_2_ asphyxiation.

### Genotyping of TRPV2 heterozygous and WT rats.

Genomic DNA was extracted from ear punch biopsies and amplified with the REDExtract-N-Amp Tissue PCR Kit (Sigma-Aldrich, XNAT-100RXN) using *Trpv2* primer mix (IDT) before being loaded on to a 2.5% agarose gel containing Midori Green dye (AnaChem). The *Trpv2* primer sequences used for genotyping were as follows: F: 5′TCTGGTTGGCTGCCTCTTAT3′ and R: 5′AGAGGCAGGATAGGGATGGT3′. Product sizes of WT and KO alleles were 444 bp and 329 bp, as expected with a 115 bp deletion. A representative genotyping gel image of DNA from ear biopsies of TRPV2 WT and heterozygous rats is shown in Supplemental Figure 3.

### Retinal arteriole isolation.

First- and second-order rat retinal arterioles were isolated in low Ca^2+^ Hank’s solution (in mM: 140 NaCl [Thermo Fisher Scientific; catalog 11904061]; 6 KCl [Melford; catalog P0515]; 5 D-glucose [Merck; catalog G7528-1KG]; 0.1 CaCl2 [Merck; cagtalog C8106-500G]; 1.3 MgCl2 [Merck, catalog M2393-100G]; and 10 HEPES [Melford; catalog B2001-1KG] [pH 7.4] with NaOH [Merck; catalog S5881-1KG-M]) as previously described (71). In brief, retinas were rapidly removed from euthanized animals, cut into 2 halves using a scalpel blade, and mechanically triturated using a fire-polished Pasteur pipette. Following centrifugation (500*g* for 30 seconds at room temperature) and trituration steps, isolated retinal arterioles contained within retinal homogenates were stored at room temperature until further use. All experiments were carried out within 8 hours of vessel isolation.

### Arteriolar pressure myography.

Pressure-induced myogenic tone was measured in isolated retinal arterioles as previously described (71). After mounting on a custom-built pressure myography system, vessels were pressurized and maintained at 40 mmHg for 15 minutes to enable the development of steady‑state myogenic tone. Vessels were viewed under a 20×, NA 0.4 objective, and images captured at a frame rate of 140 images per minute using a MCN-B013-U USB camera (Mightex). Vessel diameter measurements were made using MyoTracker software (72).

### Patch-clamp recording.

TRPV2 currents from VSMCs embedded within isolated retinal arterioles were recorded using the cell-attached configuration of the patch-clamp technique as previously described (16). In brief, retinal arteriole segments were anchored down in a glass-bottomed recording chamber on the stage of an inverted microscope (Nikon Eclipse TE2000-S; Nikon Instruments) and digested with an enzyme cocktail to remove the basal lamina and electrically isolate the VSMCs from their neighboring cells (73). Ionic currents were recorded using an Axopatch 200B patch-clamp amplifier (Molecular Devices), and pCLAMP10 was used for data acquisition and off-line analysis. Patch electrodes were pulled from borosilicate glass with tip resistances of 3–5 MΩ. Currents were sampled at 20 kHz and were low-pass filtered at 5 kHz. Stretch-activated TRPV2 currents were activated by applying negative pressure to the membrane patch at the tip of the patch electrode (16). Experiments were performed in high K^+^ pipette and bath solutions to set the resting membrane potential of the cells close to 0 mV. Recordings were made at the predicted reversal potential for Cl^–^ ions under our recording conditions (+52.3 mV) and using a pipette solution containing inhibitors of Kv (voltage-gated K^+^ channels), BK (large-conductance Ca2^+^-activated K^+^ channels), TREK-1 (2 pore domain K^+^ channels), and VGCCs (16). Stretch-activated TRPV2 current activity was integrated, and the computed net charge movement (in picoColoumbs [pC]) per second of recording was used for statistical comparisons.

### qPCR.

Retinal arterioles were lysed by Real Time Ready Cell Lysis Kit (Roche). RNA lysates were used in real-time 1-step qPCR reactions performed on a Lightcycler 480 instrument (Roche). Relative gene expression values were evaluated with the 2^–ΔΔCt^ method using *Actb* as the housekeeping gene. qPCR primers used in this study are listed in Supplemental Table 2.

### IHC of rat retinas.

Processing and immunolabeling of rat retinal whole mounts and cryosections (14 μm thick; center-middle retinal eccentricities) were performed as previously described (16, 40). Details of the primary and secondary antibodies used and their dilutions are listed in Supplemental Table 3. Cell nuclei were counterstained with either TO-PRO-3 (pseudo-colored blue; Thermo Fisher Scientific) or DAPI (Vector laboratories). For detection of isolectin binding, isolectin-B4 from *Bandeiraea simplicifolia* (1:200; biotin conjugate; Sigma-Aldrich; catalog L2140) followed by streptavidin conjugated to Alexa Fluor 488 or Alexa Fluor 568 (1:200; Thermo Fisher Scientific; catalogs S11223 and S11226, respectively) was used. Confocal images were acquired using either a Leica SP5 (Leica Microsystems) or Nikon C1 (Nikon Ltd.) laser scanning confocal microscope.

### IHC of human retinas.

Human postmortem retinas from age‑matched nondiabetic and type 2 diabetic donors (*n* = 4 per group) were obtained from the National Disease Research Interchange (NDRI; Philadelphia, Pennsylvania, USA) as previously described (74). The demographics of the sample donors are shown in Supplemental Table 4. After deparaffinization, antigen retrieval was carried out by heating in sodium citrate buffer (10 mM sodium citrate acid, 0.05% Tween 20 [pH 6.0]). After washing in PBS, sections were incubated (4°C) for 24 hours with primary TRPV2 (1:100; Merck; catalog PC421) and α-SMA–Cy3–conjugated (VSMC marker; 1:200; Sigma-Aldrich; catalog C6198) antibodies. Donkey anti–rabbit Alexa Fluor 488 was used for secondary detection of bound anti-TRPV2 antibodies. Images were acquired randomly from 4 retinal arterioles per donor using a Leica SP5 confocal laser scanning microscope.

### Rat kidney histology and IHC.

Kidneys were harvested from TRPV2 WT and heterozygous rats and were either fixed in 10% formalin for routine histology with H&E staining or snap frozen in OCT for IHC. Histological sections were digitally scanned at high resolution and imaged using PathXL software (Philips Digital Pathology). Frozen cryostat-cut sections (10 μm thick; upper pole of each kidney) were fixed with 4% PFA in PBS and colabeled with TRPV2 (1:200; Merck; catalog PC421) and pancadherin (1:100; Abcam; catalog ab6528) antibodies. TRPV2 and pancadherin were detected using donkey anti–rabbit Alexa Fluor 488 and donkey anti–mouse Alexa Fluor 568, respectively (1:200; Thermo Fisher Scientific; catalogs A21206 and A10037). For imaging, a Leica SP8 confocal laser scanning microscope (Leica Microsystems) was used.

### Analysis of IHC images.

In all IHC protocols, laser power and confocal settings were kept constant within experiments, and all images were background subtracted prior to analysis. Rat retinal images were analyzed using ImageJ (NIH) software (75) in accordance with our previously published methods (31, 40, 45, 73). Glial and neuronal cell data were acquired from 2 sections per retina obtained from central and midretinal eccentricities. Six images were captured per section, and values were averaged and normalized per 100 μm of retinal length or per unit area of retinal tissue in mm^2^. Neovascular areas were measured in the superficial vascular plexus of isolectin-B4–stained retinal whole mount preparations (5 images per retinal quadrant) and expressed as a percentage of the total retinal area analyzed. Imaris software (Bitplane AG) was used for quantifying VSMC-specific and total TRPV2-associated immunofluorescence in human retinal and rat kidney sections, respectively.

### Western blotting.

Whole retinas were lysed in RIPA buffer containing protease inhibitors (Thermo Fisher Scientific). Supernatants were cleared by centrifugation at 12,000*g* for 15 minutes (4°C), and protein concentration was determined using a BCA protein assay kit (Thermo Fisher Scientific). Protein samples (30 μg) were separated on 10% SDS polyacrylamide gels, transferred to PVDF membranes, and probed with polyclonal rabbit antibodies raised against TRPV2 (1:500; Merck; PC421) and mouse monoclonal anti–β-actin antibodies (1:5,000; Cell Signaling Technology; 8H10D10). After washing, membranes were incubated with appropriate goat anti–rabbit and anti–mouse HRP-conjugated secondary antibodies (1:2,000; Cell Signaling Technology; catalogs 7074 and 7076S). Protein bands were visualized by enhanced chemiluminescence and imaged using a Syngene G:BOX Chemi XX6system (Syngene). Immunoblots were quantified by densitometry, and TRPV2 protein expression was normalized to β-actin levels.

### Cytokine array.

The Abcam Rat Cytokine Antibody Array Kit (Abcam; ab133992), which enables the detection of 34 rat cytokines, was used to assess relative inflammatory cytokine levels in retinal samples from TRPV2 WT and heterozygous rats. Retinas were lysed with RIPA buffer and homogenized on ice. Lysates were clarified by centrifugation at 12,000*g* for 15 minutes at 4°C, and protein concentrations were determined using a BCA protein assay kit (Thermo Fisher Scientific; 2322). In total, 300 μg of protein was applied per array, and all steps in the sample analysis procedure were performed in accordance with the manufacturer’s instructions. Array images were analyzed using ImageJ software (75). Background staining was subtracted, and the relative intensity of each spot was normalized to positive control spots on each array. The integrated density of duplicated spots representing each cytokine was quantified. The cytokine array experiment was performed in triplicate with 3 different biological pools of retinas for each genotype.

### Blood pressure measurements.

Blood pressure was measured in conscious rats using tail cuff plethysmography (ML125 NIBP System; AD Instruments). Rats were allowed to habituate to the plethysmography procedure for 7 days prior to experiments being performed. Blood pressure measurements were made in triplicate for each animal, and the average systolic, diastolic, and mean arterial blood pressures were calculated.

### IOP.

IOP was determined in anesthetized rats (sodium pentobarbital, 50 mg/kg, i.p.) within 2 minutes of anesthesia induction by means of applanation tonometry (Tono-Pen AVIA Vet; Reichert Technologies). The mean of 6 consecutive IOP measurements was recorded for each eye. All measurements were performed between 10 a.m. and noon to diminish the impact of diurnal changes in IOP.

### Retinal pressure autoregulation.

Retinal blood flow autoregulation in response to an acute increase in systemic blood pressure was assessed in 3-month-old TRPV2 WT and heterozygous rats by injection of the sympathomimetic amine, tyramine (28). Rats were anesthetized with Rompun (s.c., 20 mg/kg; Bayer) and ketamine (i.p., 150 mg/kg; Ketavet; Zoetis), and their eyes were dilated with 1% atropine and 2.5% phenylephrine eye drops (Bausch & Lomb). The depth of anesthesia was assessed using toe-pinch and eye-blink reflexes. Basal blood pressure readings were taken immediately following anesthesia by tail cuff plethysmography. During all procedures, the body temperature of the rats was monitored and kept constant at 37°C with a heating pad (Kent Scientific). To raise blood pressure, tyramine was injected i.v. at a dose of 1 mg/kg (MilliporeSigma). Recordings of retinal blood flow were taken once the blood pressure increase had stabilized, typically 5 minutes after tyramine injection. RAVF was calculated as previously described using fluorescently labeled microspheres and fluorescein angiography (76, 77). Retinas were imaged using a Micron IV system (Phoenix Technologies) with the optic disc centered in the field of view. FluoSpheres (50 μL/100g body weight; 2 μm bead diameter; Thermo Fisher Scientific; catalog F8827) were i.v. injected, and fluorescence images were collected for 5 minutes at 12 fps. Once the microsphere recording was completed, 10% sodium fluorescein (25 μL/100g body weight; Sigma-Aldrich) was injected i.v. Fluorescein angiography images were captured immediately after filling of the retinal arterioles to allow their diameters to be determined prior to any significant leakage of the dye. Filling of the retinal arterioles was typically complete 40 seconds after dye injection. All analyses were performed offline. Microspheres moving through the retinal arterioles were identified by their direction of travel from the optic disc to the peripheral retina and by cross-referencing the images with those obtained from the fluorescein angiography. Microsphere streak lengths were measured in ImageJ (75) and divided by the camera exposure time to determine arteriolar flow velocities. The fluorescein signal in the retinal arterioles was 1.5- to 2-fold higher than that of the choroidal and retinal background, allowing the arteriole diameters to be easily discerned. Arteriole diameters were determined from measurements at 5 equidistant locations along the vessel length using the line tool in ImageJ. Arteriolar volumetric flow was calculated as the product of the average microsphere velocity and the arteriole cross-sectional area as previously described (77). Separate groups of TRPV2 WT and heterozygous rats were used to measure basal arteriolar volumetric flow for comparison with the tyramine-injected animals. For the basal flow measurements, animals were i.v. injected with PBS at an equivalent volume to that used for the tyramine injections. This experimental design was necessary, since repeated use of the microsphere method was not possible within a given recording session, and procedures were licensed under nonrecovery anesthesia, precluding an assessment of blood flow in the same animal before and after tyramine injection.

### Electroretinography.

Scotopic ERGs were recorded and analyzed using a Espion Visual Electrophysiology System (Diagnosys Technologies). Rats were dark adapted overnight and anesthetized with Rompun (s.c., 20 mg/kg) and ketamine (i.p., 150 mg/kg), and the pupils were dilated with 1% atropine and 2.5% phenylephrine eye drops. ERGs were recorded using rodent corneal electrodes (Diagnosys Technologies), with the reference and ground electrodes placed s.c. in the forehead and tail, respectively. The amplitudes and implicit times for the a- and b-waves were measured as a function of increasing light intensity (0.008–25 cd·s/m^2^). ERG signals were averaged from 5 responses at each intensity level, with an interstimulus interval of 10 seconds (0.008, 0.025 cd·s/m^2^), 15 seconds (0.08, 0.25, 0.8 cd·s/m^2^) or 30 seconds (2.5, 8, 25 cd·s/m^2^). OPs were obtained by low band filtering at 75 Hz and recorded using 25 cd·s/m^2^ flash intensity. Summed OP amplitudes and implicit times were calculated from wavelets 2–5.

### Pupil diameters.

Pupil diameters were measured in anesthetized rats (sodium pentobarbital, 50 mg/kg, i.p.) from anterior segment images taken using a Micron IV system in bright-field mode. For each animal, measurements were taken from both eyes prior to and 5 minutes after application of mydriatics (1% atropine and 2.5% phenylephrine). Caliper measurements of the full eye diameter were used to calibrate the images. Pupil measurements were made using ImageJ.

### Retinal vasopermeability.

Blood-retinal barrier disruption was assessed by measuring albumin leakage into the retina using the Evans blue assay in accordance with published protocols (29). Briefly, rats were anesthetized with isoflurane and Evans blue dye (30 mg/mL in PBS; Sigma-Aldrich) administered i.v. by tail vein injection at a dose of 45 mg/kg. Rats were then recovered from anesthesia, and the dye was allowed to circulate for 2 hours prior to euthanasia using sodium pentobarbital. An intracardiac blood sample was collected, and the animals were perfused with citrate buffer (pH 3.5) through the left ventricle for 5 minutes at a pressure of 70 mmHg. Retinas were then harvested, and their wet weights were determined. After freeze drying, retinal dry weights were measured, and Evans blue dye was extracted in formamide at 70°C for 18 hours. he extracts were clarified by centrifugation (10,000*g* for 10 minutes at room temperature) and read spectrophotometrically at 620 nm and 740 nm. The concentration of dye in the extracts was calculated from a standard curve of Evans blue in formamide. Evans blue leakage was determined using the formula described by Qaum et al. (29).

### Statistics.

Data are presented as box-and-whisker plots showing the median, interquartile ranges (IQRs), and minimum and maximum values, with “+” in Figure 1B and Figure 4D indicating the mean. Statistical analyses were performed using Prism V8.4.3 (GraphPad). All data were checked for normality using the Shapiro-Wilk test. Normally distributed data were compared with 2-tailed Student’s *t* tests or ANOVA (1-way repeated-measures or 2-way) with Bonferroni’s post hoc correction for multiple comparisons. Nonnormally distributed data were analyzed using nonparametric Mann–Whitney *U* tests or log transformed prior to conducting 2-way ANOVA. In all experiments, a *P* value of 0.05 was considered statistically significant. *P* < 0.05 was considered significant. In figure captions, all reported *P* values for the ANOVA tests refer to post hoc comparisons. Fold and percentage changes reported in the paper are based on mean values.

### Study approval.

The study was approved by the Research Ethics Committee at Queen’s University Belfast and was conducted in accordance with the principles of the Declaration of Helsinki. All animal experiments were conducted in accordance with the Association for Research in Vision and Ophthalmology (ARVO) Statement for the Use of Animals in Ophthalmic and Vision Research and were approved by the Queen’s University of Belfast Animal Welfare and Ethical Review Body. Work adhered to Department of Health, Social Services and Public Safety (DHSSPS) project licenses PPL2786 and PPL2888.

## Author contributions

TMC conceived the project with input from MO, MKM, AWS, and JGM. MO, GE, MKM, JMRH, DPB, and TMC designed the experiments. MO, GE, MKM, JMRH, JA, P. Canning, KSE, PB, TF, P. Cincolà, JH, KM, HF, DPB, DJG, and TMC performed or supervised experiments. MO, GE, MKM, JMRH, JA, PB, TF, P. Cincolà, JH, KM, HF, and TMC analyzed the data. TJL contributed with human retinal samples. TMC and MO wrote and edited the manuscript, with input from all other authors. All authors read and approved the final manuscript for submission.

## Supplementary Material

Supplemental data

## Version 1 09/22/2022

Electronic publication
