# Supplementary material for: Loss of TRPV2-mediated blood flow autoregulation recapitulates diabetic retinopathy in rats
Source: JCI Insight. 2022 Sep 22;7(18):e155128. doi: 10.1172/jci.insight.155128 (PMC9675469; doi:10.1172/jci.insight.155128)
Supplement: Supplemental data [file jciinsight-7-155128-s145.pdf]

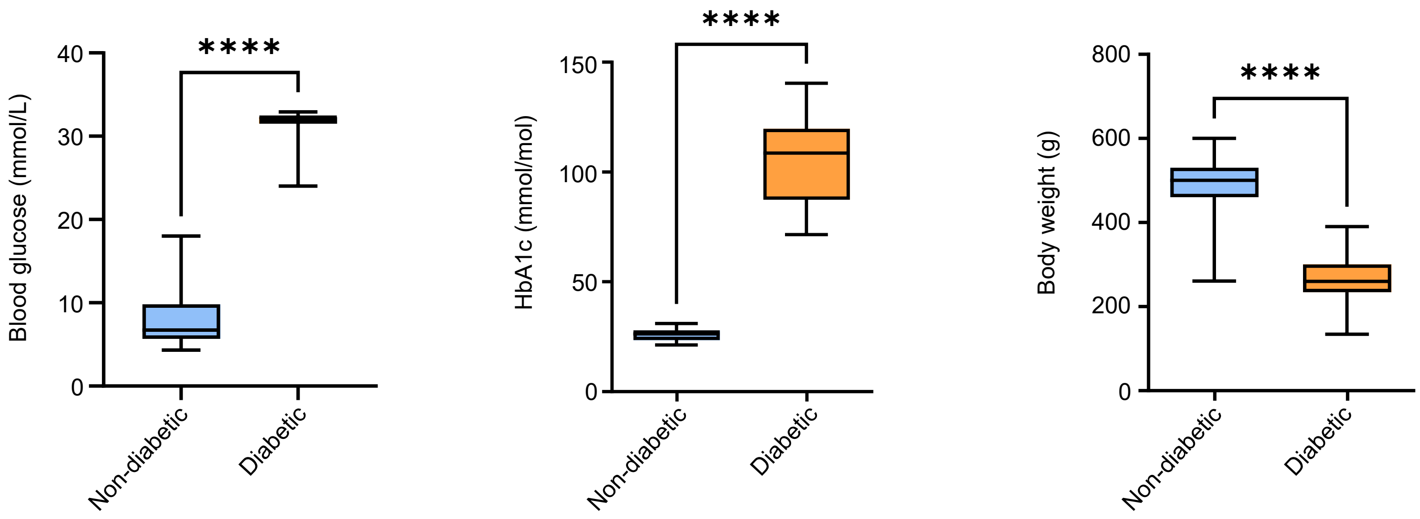

Supplemental Figure 1. Characteristics of the non-diabetic and diabetic rats. Box-and-whisker plots (min, max, 25th–75th percentile, median) comparing blood glucose, HbA1c and body weights of the diabetic rats with those of the non-diabetic controls. Data information: ****P<0.0001 based on Mann–Whitney U-test; N=69-96 animals per group for weights, N=72-74 animals per group for blood glucose and N=12-13 animals per group for HbA1c.

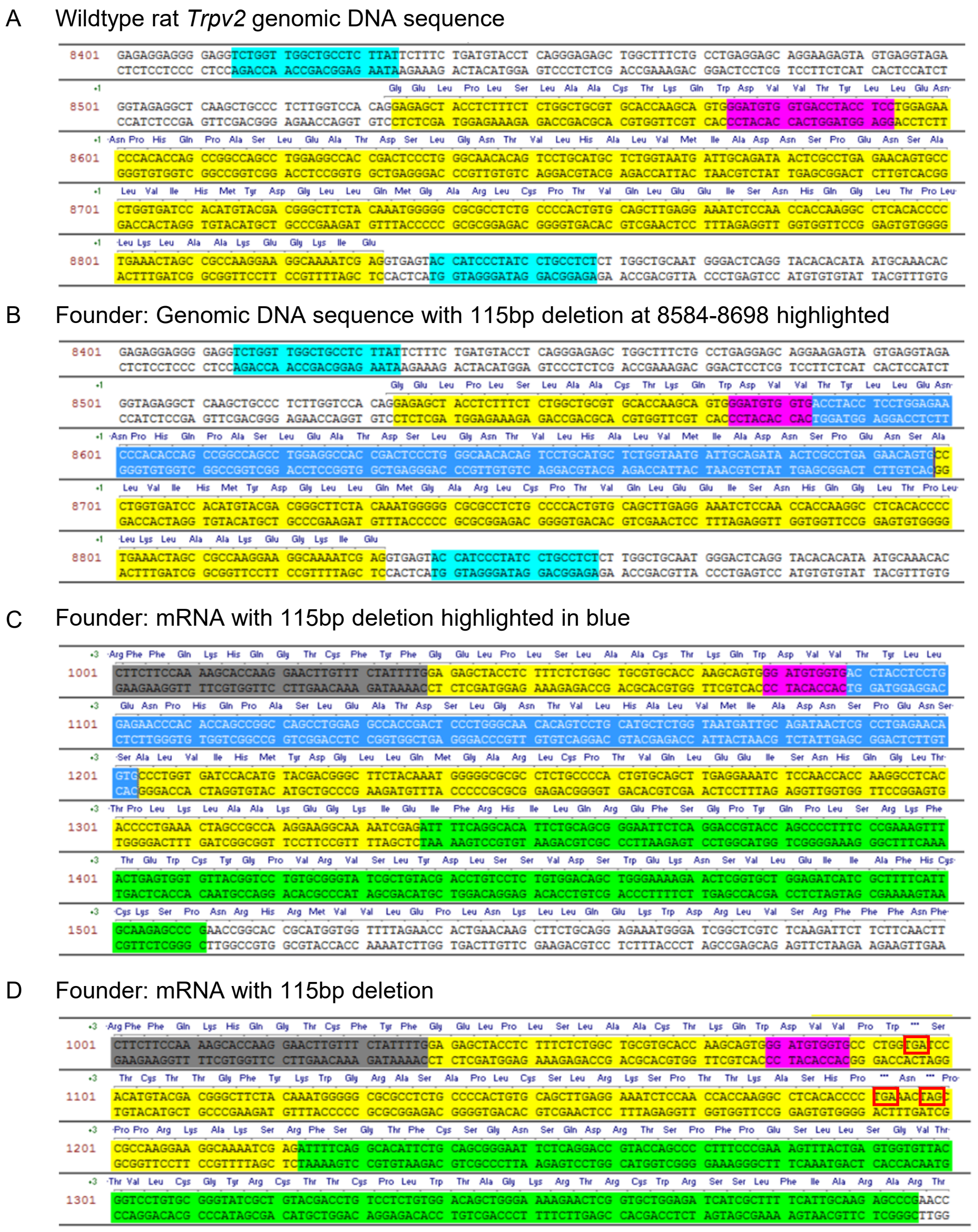

Supplemental Figure 2. Generation of TRPV2 heterozygous knockout rats using CRISPR/Cas9 genome editing. A. Part of the genomic DNA sequence of the wildtype rat *Trpv2* gene. Exon 5 is shown in yellow; the guide RNA (gRNA) site selected to target Exon 5 is coloured pink and the primer positions used for genotyping analysis are shown in cyan. B. The founder animal had a 115bp deletion at 8584-8698 of the *Trpv2* genomic DNA sequence (highlighted in blue). C. Wildtype mRNA sequence (top strand) with the deletion mutation highlighted in blue. Grey is exon 4, yellow is exon 5 and green is exon 6. D. mRNA sequence (top strand) after the deletion of the sequence highlighted in C. The 115bp deletion resulted in several early stop codons highlighted by the red boxes, the first of which was 57 nucleotides downstream of the start of exon 5.

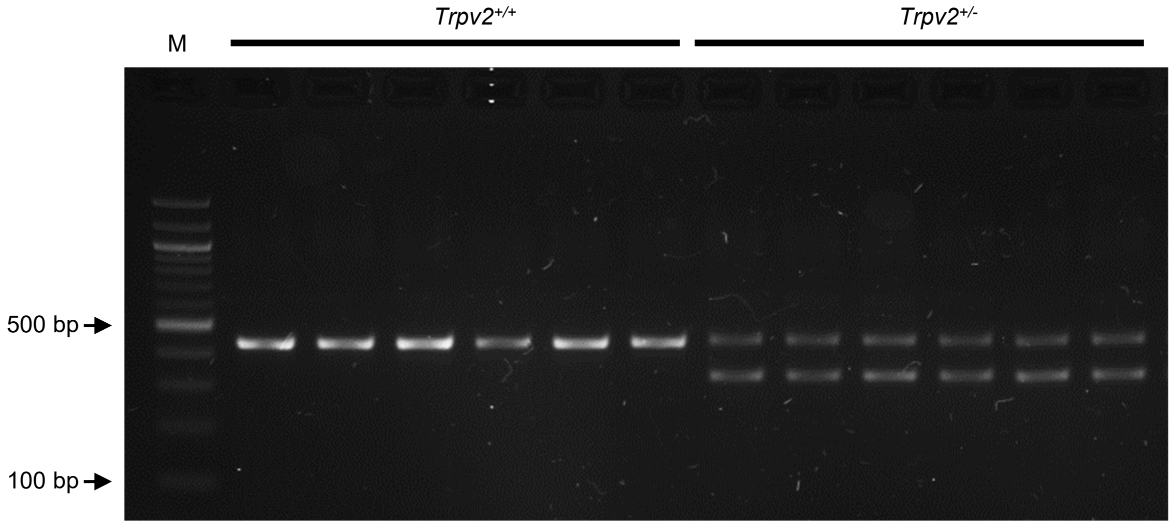

Supplemental Figure 3. Genotyping of TRPV2 heterozygous knockout rats. Representative genotyping gel of DNA from ear biopsies of TRPV2 WT and heterozygous knockout rats. Each lane represents an individual animal. TRPV2 WT rats showed a single band at 444bp, whereas the TRPV2 heterozygous knockout rats had an additional band at 329bp. The lengths of the PCR products were as expected based on the primer sets used (see Methods). “M” refers to the molecular weight marker lane.

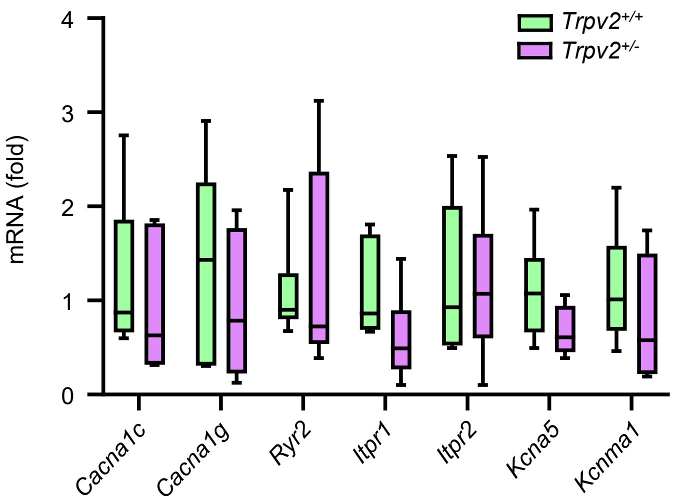

Supplemental Figure 4. Relative transcript expression for other ion channels involved in the retinal myogenic response. Box-and-whisker plots (min, max, 25th–75th percentile, median) showing that the mRNA expression levels of Ca^{2+} (*Cacna1c, Cacna1g, Ryr2, Itpr1, Itpr2*) and K^+ (*Kcna5, Kcnma1*) channels previously implicated in retinal myogenic function were similar in isolated retinal arterioles from TRPV2 WT and heterozygous rats. Data information: NS for each gene based on Mann–Whitney U-tests; N=6 animals per group.

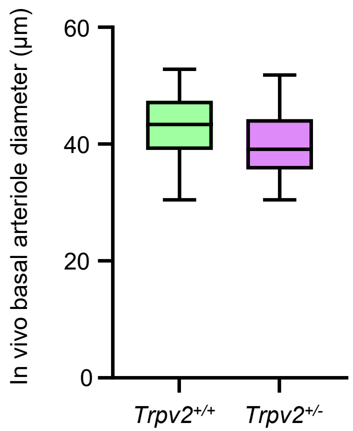

Supplemental Figure 5. In vivo basal retinal arteriole diameters in TRPV2 WT and heterozygous rats. Box-and-whisker plots (min, max, 25th–75th percentile, median) showing that in vivo retinal arteriole diameters were similar for both groups of animals under basal conditions. Data information: NS based on Mann–Whitney U-test; N=6 animals, *n*=19-23 arterioles per group.

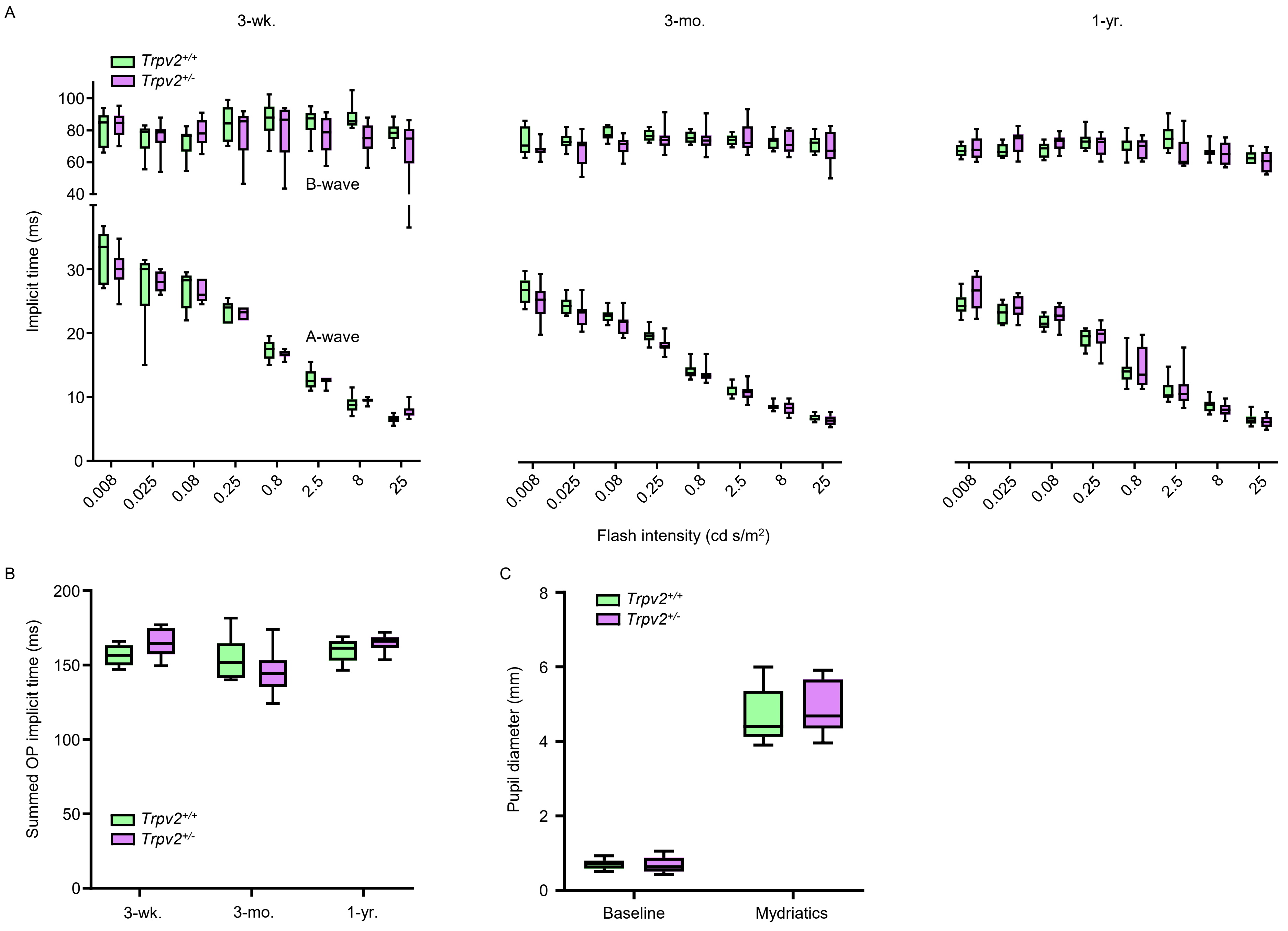

Supplemental Figure 6. ERG implicit times and pupil diameters in TRPV2 WT and heterozygous rats. A. Box-and-whisker plots (min, max, 25th–75th percentile, median) comparing ERG A- and B-wave implicit times for TRPV2 WT and heterozygous rats at 3-weeks, 3-months and 1-year of age. Data information: NS based on two-way ANOVA; N=6-7 animals per group. B. Summary data showing that summed oscillatory potential implicit times were similar between TRPV2 WT and heterozygous rats at all timepoints examined. Data information: NS based on two-way ANOVA; N=6-7 animals per group. C. Pupil diameters in TRPV2 WT and heterozygous rats (3-6 months of age) in the absence or presence of mydriatics (1% atropine and 2.5% phenylephrine). Data information: NS based on Mann–Whitney U-tests; N=7-8 animals per group.

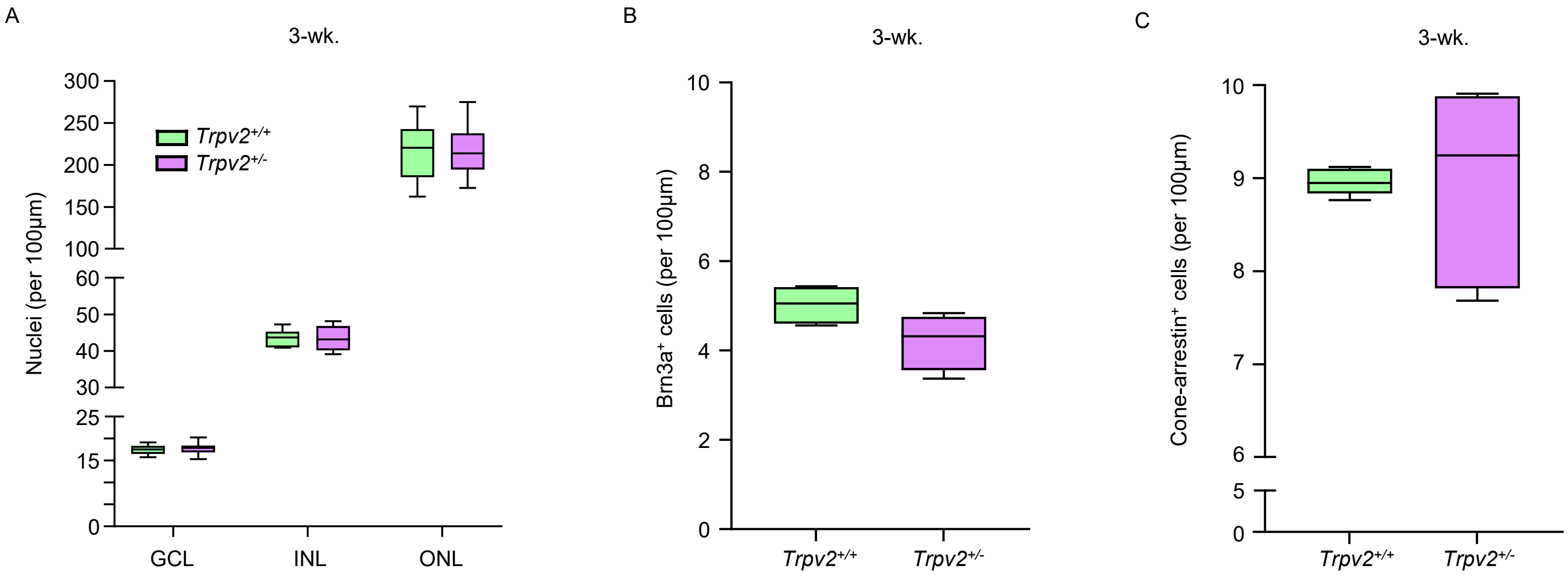

Supplemental Figure 7. Analysis of cell nuclei, ganglion cell and cone cell numbers in retinal sections from 3-week-old TRPV2 WT and heterozygous rats. A. Box-and-whisker plots (min, max, 25th–75th percentile, median) showing the numbers of cell nuclei in the ganglion cell layer (GCL), inner nuclear layer (INL) and outer nuclear layer (ONL) of 3-week-old TRPV2 WT and heterozygous rats. Data information: NS based on Mann–Whitney U-tests; N=12 animals, n=12 retinas per group. B. Summary data showing the numbers of Brn3a positive retinal ganglion cells in TRPV2 WT and heterozygous rats at 3-weeks of age. Data information: NS based on Mann–Whitney U-test; N=4 animals, *n*=4 retinas per group. C. Pooled data showing that cone cell numbers were similar in TRPV2 WT and heterozygous rats at 3-weeks of age. Data information: NS based on Mann–Whitney U-test; N=5 animals, *n*=5 retinas per group.

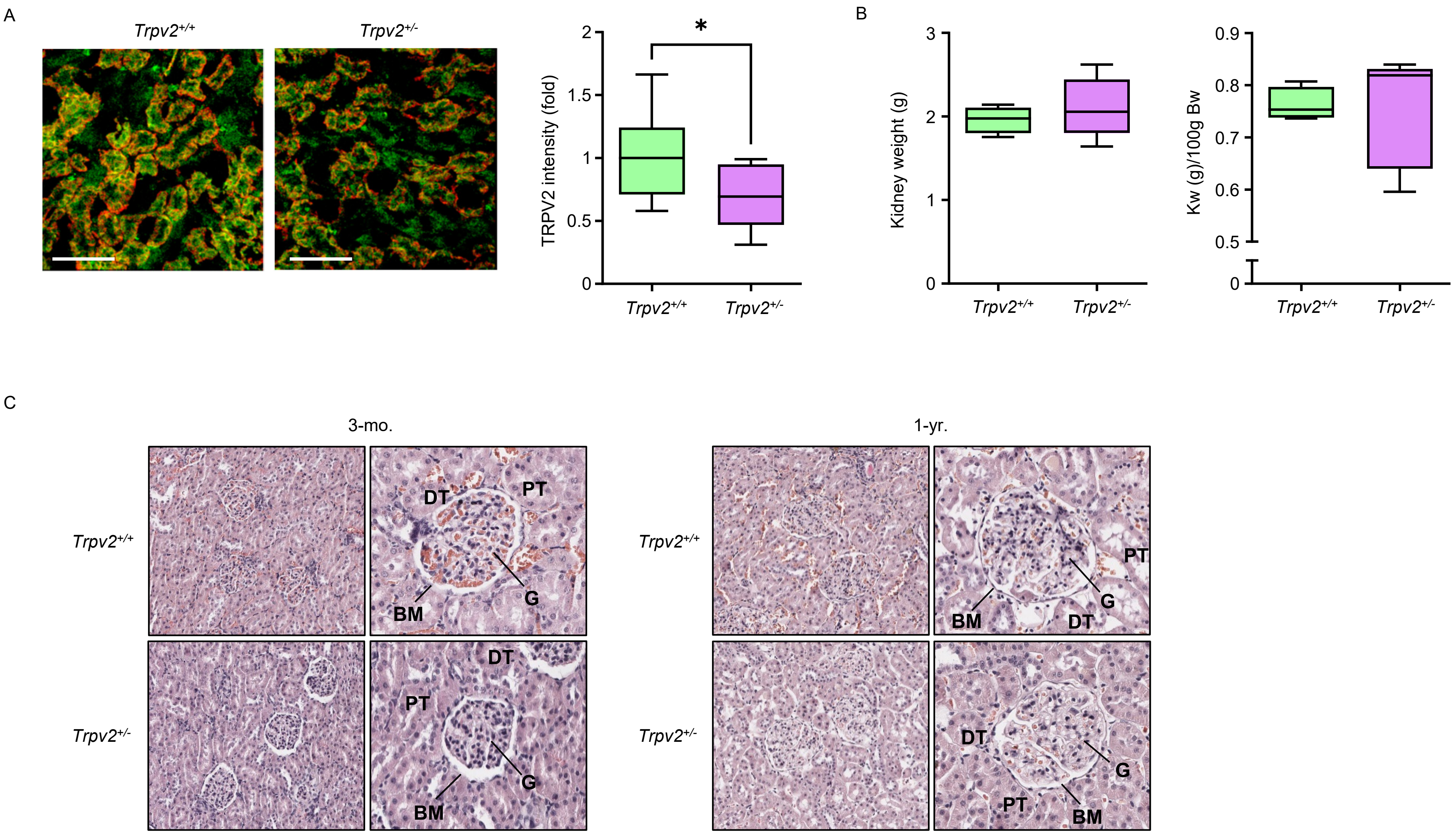

Supplemental Figure 8. Kidney structure is normal in TRPV2 heterozygous rats. A. Left, representative confocal images showing that TRPV2 protein levels (green) were reduced in the kidneys of TRPV2 heterozygous rats at 3-months of age. Kidney sections were co-labelled with anti-pan-cadherin antibodies (red) to mark the renal tubular epithelial cells. Scale bars = 100μm. Right, TRPV2 fluorescence intensity in kidney sections from TRPV2 heterozygous rats expressed relative to TRPV2 WT controls. Data information: *P<0.05 based on Mann–Whitney U-test; N=3 animals, *n*=9 renal sections per group. B. Box-and-whisker plots (min, max, 25th–75th percentile, median) comparing kidney weights and kidney weights (Kw) adjusted for body weight (Bw) between TRPV2 WT and heterozygous rats at 3-months of age. Data information: NS based on Mann–Whitney U-test; N=5 animals per group C. Representative photomicrographs of H&E stained kidney sections from TRPV2 WT and heterozygous rats at 3-months and 1-year of age. Renal morphology appeared normal in both groups of animals. G – glomerulus, BM – basement membrane, DT – distal tubule. PT – proximal tubule.

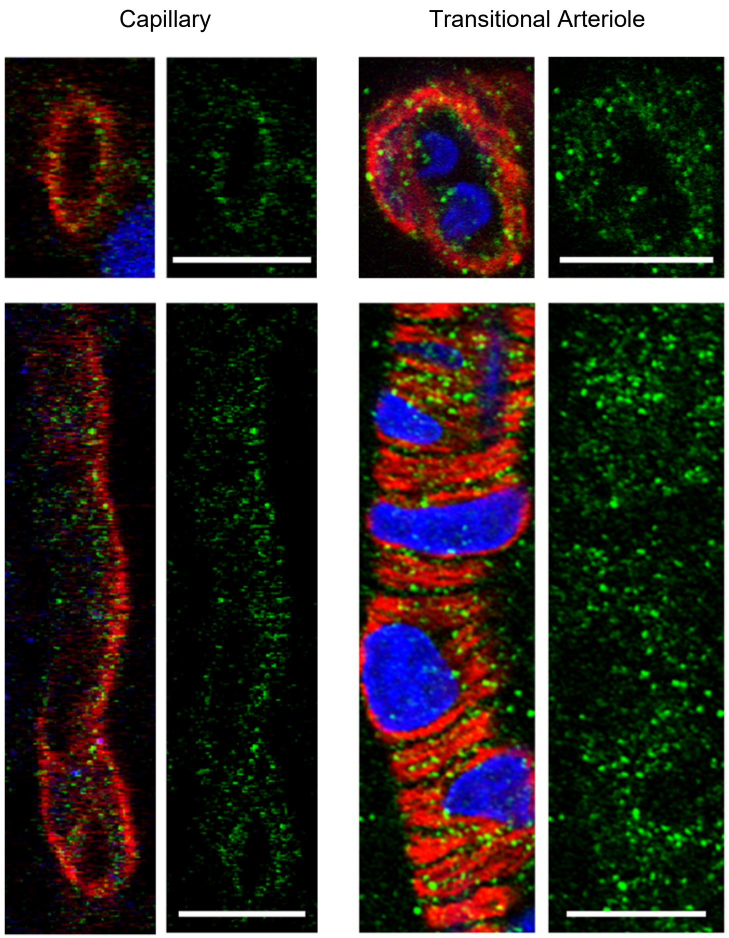

Supplemental Figure 9. TRPV2 protein expression in a retinal capillary pericyte and VSMCs of a transitional retinal arteriole. Left, confocal cross-sectional (top) and en face images (bottom) from a rat retinal wholemount preparation showing TRPV2 protein expression (green) in an NG2-labelled retinal capillary pericyte (red). Blue staining in the cross-sectional image represents a BRN3a-positive RGC. Right, Equivalent confocal images to those on the left, but showing TRPV2 protein expression (green) in α-SMA-labelled VSMCs (red) of a transitional retinal arteriole. Cell nuclei are labelled using TO-PRO-3 (pseudo-colored blue). Scale bars in all images = 15µm.

Inflammatory factor	Fold change *Trpv2*$^{+/-}$ vs *Trpv2*$^{+/+}$	Adjusted P
Activin A	1.33	P>0.05
Agrin	1.56	P>0.05
B7-2/CD86	0.94	P>0.05
B-NGF	0.95	P>0.05
CINC-1	1.17	P>0.05
CINC-2a	1.29	P>0.05
CINC-3	1.66	P>0.05
CNTF	1.86	P>0.05
Fas-Ligand	1.77	P>0.05
Fractalkine	2.58	P>0.05
GM-CSF	4.39	P<0.05
ICAM-1	5.31	P<0.0001
IFN-y	3.28	P>0.05
IL-1a	2.66	P>0.05
IL-1B	2.12	P>0.05
IL-1R6	2.57	P>0.05
IL-2	2.04	P>0.05
IL-4	1.90	P>0.05
IL-6	1.87	P>0.05
IL-10	1.69	P>0.05
Il-13	3.69	P<0.05
Leptin	4.63	P>0.05
LIX	4.27	P>0.05
L-Selectin	4.70	P>0.05
MCP-1	3.29	P>0.05
MIP-3a	3.31	P>0.05
MMP-8	2.34	P>0.05
PDGF-AA	3.17	P>0.05
Prolactin R	3.07	P>0.05
RAGE	1.28	P>0.05
CXCL7	2.13	P<0.05
TIMP-1	1.70	P>0.05
TNF-a	3.40	P<0.05
VEGF	2.65	P>0.05

Supplemental Table 1. Summary of rat cytokine array results. The Abcam Rat Cytokine Antibody Array was used to simultaneously detect the levels of 34 cytokines in retinal samples from TRPV2 WT and heterozygous rats. Relative quantification of spot intensities between the two groups was performed using Image J. Statistical comparisons are based on two-way ANOVA; blots were performed in triplicate with three different biological pools of retinas for each genotype; N=18 animals per group in total, N=6 animals per replicate.

Gene	Forward Primer	Reverse Primer
Actb	TGCCCTAGACTTCGAGCAAG	GGCAGCTCATAGCTCTTCTCC
Trpv2	GACCTCCTAAAAACACTTCTGCTC	AGAGTCGGTCACGGTCAAAC
Cacna1c	GGAGGCAGAATGCAAGGGTA	TGTTCTCCCAACTTCGAGGC
Cacna1g	CTTCCAGGACAGGTGGAACC	GGAGCACCCTCATGATACGG
Ryr2	ATCCCAACGCAGCAAGGAAA	TTCACCTTTGCTGGCACTGA
Itpr1	GCTGGCTGCTGTGGGTTGAC	CCCAAAGAAGAGCTGCTCCC
Itpr2	CAGTGCGGTGGCATGTGATG	CAGAACTCCAGTCACAGG
Kcna5	CAGGACCAGCCAAGGATCG	TTCCCACACCCCCAACTCA
Kcnma1	GGCGGCTGATCTATTCCAAGAT	CTCAGAACGTCCGCAATCAA

Supplemental Table 2. Primer sequences used for RT-qPCR. All primers were purchased from Integrated DNA Technologies (Coralville, IA, USA), with the exception of those used in Fig 2A, which were from Roche (Roche, Basel, Switzerland).

Target	Antibody	Company – Cat No.	Dilution
TRPV2	Rabbit polyclonal	Merck (PC421)	1:100
α-SMA	Mouse monoclonal Cy3 conjugated	Sigma (C6198)	1:200
Albumin	Goat polyclonal	Bethyl (A90-134A)	1:1000
Collagen IV	Rabbit polyclonal	Bio-Rad (2150-1470)	1:200
RUNX1	Sheep polyclonal	Lifespan (LS-C94757)	1:100
GFAP	Rabbit polyclonal	Dako (N1506)	1:200
Vimentin	Mouse monoclonal	Sigma (V6630)	1:200
Iba1	Rabbit polyclonal	Wako (019-19741)	1:500
Brn3a	Goat polyclonal	Santa Cruz (sc-31984)	1:200
Cone arrestin	Rabbit polyclonal	Merck (AB15282)	1:500
GABA	Rabbit polyclonal	Sigma (A2052)	1:500
**Secondary Antibody**		**Company – Cat No.**	**Dilution**
Donkey anti-rabbit Alexa Fluor-488		ThermoFisher (A21206)	1:200
Donkey anti-goat Alexa Fluor-488		ThermoFisher (A11055)	1:200
Donkey anti-sheep Alexa Fluor-405		Abcam (ab175676)	1:200
Donkey anti-mouse Alexa Fluor-568		ThermoFisher (A10037)	1:200

Supplemental Table 3. Primary and secondary antibodies used for the immunolabelling of rat retinal wholemount preparations and cryosections.

Donor	Sex	Age
Non-diabetic	Male	44 years
Non-diabetic	Male	87 years
Non-diabetic	Male	68 years
Non-diabetic	Female	72 years
Type 2 diabetes	Male	56 years
Type 2 diabetes	Female	59 years
Type 2 diabetes	Male	64 years
Type 2 diabetes	Female	70 years

Supplemental Table 4. Demographics of the non-diabetic and diabetic human sample donors. The mean age (± standard deviation) of the non-diabetic and diabetic donors was 67.8 (± 17.8) and 62.3 (± 6.1) years, respectively. Data information: NS based on Student' t test.
